# Modular Operator for Null Plane Algebras in Free Fields

**DOI:** 10.1007/s00220-022-04432-8

**Published:** 2022-09-03

**Authors:** Vincenzo Morinelli, Yoh Tanimoto, Benedikt Wegener

**Affiliations:** Dipartimento di Matematica, Università di Roma Tor Vergata, Via della Ricerca Scientifica 1, 00133 Rome, Italy

## Abstract

We consider the algebras generated by observables in quantum field theory localized in regions in the null plane. For a scalar free field theory, we show that the one-particle structure can be decomposed into a continuous direct integral of lightlike fibres and the modular operator decomposes accordingly. This implies that a certain form of QNEC is valid in free fields involving the causal completions of half-spaces on the null plane (null cuts). We also compute the relative entropy of null cut algebras with respect to the vacuum and some coherent states.

## Introduction

The modular Hamiltonian, or the (logarithm of the half of the) modular operator of local regions in quantum field theory (QFT), has been a focus of attention in recent years (see e.g. [CF20, Lon20, CTT17b]). On one hand, quantum-information aspects, such as the Bekenstein bound, generalized second law of thermodynamics and various null energy conditions, are a rare guidepost in the search of quantum gravity [Cas08, Wal12, FLPW16]. On the other hand, the modular theory of von Neumann algebras allows one to define relative entropy in QFT in a mathematically precise way [OP04], and various modular objects in QFT have been computed in concrete examples [LX18, Hol20, CLR20]. In particular, the modular operator of certain regions in the null plane $X_-^0:=\{x=(x_0,\ldots ,x_d)\in {{\mathbb {R}}}^{D+1}:x_0-x_1=0\}$ (or its Poincaré transformed) has played an important role in relation with the quantum null energy condition (QNEC) and the averaged null energy condition (ANEC) [CTT17b, KLLSM18, CF20]. In these works, physicists consider a null cut, a region in the null plane $X_-^0$ defined by a spacelike curve *C*, and have written a formula for the modular operator for the algebra of a null cut (see e.g. [CTT17b,  (1.5)]):

1 $$\begin{aligned} {\hat{H}}_C = 2\pi \int d^{D-1}\pmb {x}_\perp \int _{-\infty }^\infty d\lambda (\lambda - C(\pmb {x}_\perp ))T_{++}(\lambda ,\pmb {x}_\perp ), \end{aligned}$$

where $T_{++}$ is the lightlike component of the stress-energy tensor. This suggests that the inclusion of null cut algebras is a half-sided modular inclusion (HSMI) [Wie93]. Based on the latter assumption, a limited version of QNEC has been proved in [CF20]. Therefore, it is crucial to study modular objects on the null plane.

Actually, the above formula must be interpreted with care: while it seems reasonable to assume that the stress-energy tensor is an operator-valued distribution (or even a Wightman field), it is unclear whether it can be restricted to a null plane (cf. [Ver00, FR03]). Furthermore, it is integrated against the unbounded function $\lambda - C(\pmb {x}_\perp )$, that could be even more problematic. For these reasons, (1) cannot be considered directly as an expression for an operator on a Hilbert space. One of the goals of this paper is to partially justify (1) using the modular theory of von Neumann algebras in the case of the free fields.

We observe that the (scalar) free field can be restricted to the null plane, through a corresponding condition on the test functions. This has been known for a long time, and general properties of observables on the null plane have been studied [SS72, Dri77b, Dri77a, GLRV01, Ull04]. By the Bisognano–Wichmann property [BW76] and the Takesaki theorem [Tak03,  Theorem IX.4.2], it is immediate that, if there are enough observables on the null plane, they split into a (continuous) tensor product along the transverse direction. This allows us to consider the observables on each fibre on the null plane. These observables form a simplified quantum field theory on each lightlike fibre, and we can consider the modular objects there. We will show that the one-particle subspace ${\mathfrak {H}}_m$ of the free field with mass *m* disintegrates as follows

2 $$\begin{aligned} {\mathfrak {H}}_m=\int _{{{\mathbb {R}}}^{D-1}}^{\oplus } {{\mathcal {H}}}_{U(1)}d\pmb {x}_\perp . \end{aligned}$$

where ${{\mathcal {H}}}_{U(1)}$ is the one-particle space of the *U*(1)-current sitting on the light ray $\{(t,t,\pmb {x}_\perp )\in {{\mathbb {R}}}^{1+D}:t\in {{\mathbb {R}}}\}$. One can deduce that the modular operator of the region on the null plane is decomposed into the fibres, and its logarithm is written as a direct integral over fibres (see (19)):

$$\begin{aligned} \log (\Delta _{H(N_C)})\simeq \int ^\oplus _{{{\mathbb {R}}}^{D-1}}\left( \log (\Delta _{H_{U(1)}({{\mathbb {R}}}_+)})+ 2\pi C(\pmb {x}_\perp ) P_{\pmb {x}_\perp }\right) \,d\pmb {x}_\perp , \end{aligned}$$

This is a clear analogue of (1), where the logarithm of the modular operator is expressed as the integral in the transverse direction of the stress-energy tensor smeared by the function $\lambda - C(\pmb {x}_\perp )$, where the latter is a formal expression for the shifted dilation operator in two-dimensional conformal field theory, which should coincide with the modular operator on each fibre. Our formula, while we avoid talking about the stress-energy tensor, makes explicit the idea that the modular operator decomposes into fibres. While the validity of (1) is believed more generally, we point out that in general there are not many observables that can be restricted to the null plane. We clarify the situation from interacting models in $(1+1)$-dimensions. Our formula allows a covariant action of such distorted dilations of the null plane and, for null cuts with continuous boundary *C*, on distorted wedge region $W_C=N_C''$.

In the course of the proof (Proposition 4.1), we show that inclusions of the null cut regions are HSMI. Together with [CF20], this completes the limited version of QNEC as in (25) in the case of the free scalar field (note that the result of [CF20] is based on the assumption that these inclusions are HSMI).

Entropy inequalities can be used to investigate features of quantum systems. For instance, on the physical grounds, the strong subadditive property of the entropy together with the Lorentz covariance leads to a *c*-theorem for the entanglement entropy in 1+1 dimensions and connections with the *a*-theorem are claimed in [CTT17a]. Due to the direct integral disintegration of the one-particle space (2), it is possible to generalize the Buchholz–Mach–Todorov endomorphism $\beta _k$ [BMT88] to the direct integral of the *U*(1)-current with $k\in C_0^\infty (X^0_-)$. Using the formula contained in [Lon20], we are able to compute the relative entropy with respect to the states $\omega \circ \beta _k$ and $\omega$ and deduce the QNEC - in this case intended to be $S''(t)>0$ where *S* is the relative entropy related of the algebra ${{\mathcal {A}}}(N_{C+tA})$ with respect to $\omega \circ \beta _k$ and $\omega$ and the derivative is with respect to *t*. We also have a saturation of the strong superadditivity condition of the relative entropy considered.

This paper is organized as follows. In Sect. 2 we collect the basic notions such as one-particle space in terms of standard subspaces and its second quantization. In Sect. 3 we discuss observables on the null plane and their transversal decomposition. In Sect. 4 we obtain the decomposition of the modular operator of null plane regions and prove that inclusions of null plane regions are HSMI. In Sect. 5, after recalling the notions concerning relative entropy, ANEC, QNEC and some background also from physics, we study the relative entropy and its relation with the energy inequalities and the saturation of the strong superadditivity condition of the null cut algebras between the BMT type states. In Sect. 6 we present concluding remarks, including the 1+1 dimensional case.

## Preliminaries

In this Section we will recall the operator-algebraic formulation of the free field. A free field is constructed starting from its one-particle structure. A quantum and relativistic particle species on Minkowski spacetime corresponds to an irreducible unitary positive energy representation of the Poincaré group. The localization property of the one-particle states is formulated in terms of the standard subspaces, and it translates to the localization property of the associated free fields through the second quantization. We will further comment on the *U*(1)-current model, which will be a convenient tool to describe the restriction of the free theory to the null plane.

### Abstract one-particle structure

A real linear, closed subspace *H* of a complex Hilbert space ${{\mathcal {H}}}$ is called **cyclic** if $H+iH$ is dense in ${{\mathcal {H}}}$ and **separating** if $H\cap i H=\{0\}$. A **standard subspace** is a real linear, closed subspace that is both cyclic and separating. We recall below some useful properties of standard subspaces, see [Lon08] for details.

It is possible to consider an analogue of the Tomita-Takesaki modular theory for standard subspaces. For a standard subspace *H*, the Tomita operator $S_H$ is defined to be the closed anti-linear involution with dense domain $H+iH$ acting in the following way:

$$\begin{aligned} S_H: H+iH&\rightarrow H+iH \\ \xi +i\eta&\mapsto \xi -i \eta . \end{aligned}$$

The polar decomposition

$$\begin{aligned} S_H=J_H \Delta _H^\frac{1}{2} \end{aligned}$$

defines the modular operator $\Delta _H$ and the modular conjugation $J_H$, and they satisfy the following relations:

$$\begin{aligned} J_H\Delta _H J_H= \Delta _H^{-1},\ \ \Delta _H^{it}H=H \quad \text {for } t\in {{\mathbb {R}}}, \ \ J_H H=H', \end{aligned}$$

where $H'$ is the symplectic complement of *H*:

$$\begin{aligned} H':=\{\xi \in {{\mathcal {H}}}: \mathrm {Im}\,\langle \xi ,\eta \rangle =0\ \text { for } \eta \in H\}. \end{aligned}$$

The symplectic complement $H'$ is a standard subspace if and only if so is *H*, and a standard subspace *H* satisfies $H=H''$. The Tomita operator of the symplectic complement $H'$ is given by

$$\begin{aligned} S_{H'}=S_H^* = J_H \Delta _H^{-\frac{1}{2}} = \Delta _H^{\frac{1}{2}} J_H. \end{aligned}$$

There is a $1-1$ correspondence $H{\mathop {\leftrightarrow }\limits ^{1:1}}S_H$ between standard subspaces and closed, anti-linear, densely defined involution: For such an operator *S*, the real closed subspace $\ker (1-S)$ is a standard subspace.

One can easily deduce the covariance of standard subspaces (see [Mor18,  Lemma 2.2]):

#### Lemma 2.1

Let $H\subset {{\mathcal {H}}}$ be a standard subspace and *U* be a unitary or anti-unitary operator on ${{\mathcal {H}}}$. Then *UH* is standard and $U\Delta _HU^*=\Delta _{UH}^{\epsilon (U)}$ and $UJ_HU^*=J_{UH}$ where $\epsilon (U)=1$ if U is unitary and $\epsilon (U)=-1$ if *U* is anti-unitary.

The following is an analogue of Borchers theorem [Bor92, Flo98] for standard subspaces, see [Lon08,  Theorem 3.15].

#### Theorem 2.2

Let *H* be a standard subspace of a Hilbert space ${{\mathcal {H}}}$ and *T* a one-parameter group with positive generator such that $T(s)H\subset H, \ s\ge 0$, then the following hold:

$$\begin{aligned} \Delta _H^{it}T(s)\Delta _H^{-it}&=T(e^{-2\pi t}s)\\ J_H T(s) J_H&= T(-s). \end{aligned}$$

We say that a pair of standard subspaces $K\subset H$ is a **half-sided modular inclusion (HSMI)** if

$$\begin{aligned} \Delta _{H}^{-it}K\subset K \ \text { for } t\ge 0. \end{aligned}$$

Let ${\mathbf {P}}$ be the translation-dilation group, that is, the group of affine transformations of ${{\mathbb {R}}}$, where dilations act by ${\mathfrak {d}}(2\pi t) x=e^{2\pi t}x, x \in {{\mathbb {R}}}$ and translations act by ${\mathfrak {t}}(s) x = x + s$. The group ${\mathbf {P}}$ contains also dilations centered at 1: ${\mathfrak {d}}_1(2\pi t)x=e^{2\pi t}(x-1)+1$. A HSMI of von Neumann algebras implies the existence of a one-parameter group of unitaries with certain properties [Wie93, AZ05]. The following is its standard subspace version and the first part can be found in [Lon08,  Theorem 3.21].

#### Theorem 2.3

Let $K\subset H$ be a half-sided modular inclusion of standard subspaces of the Hilbert space ${{\mathcal {H}}}$, then there exists a positive energy representation of the translation-dilation group ${\mathbf {P}}$ given by

$$\begin{aligned} U({\mathfrak {d}}(2\pi t))=\Delta _H^{-it},\qquad U({\mathfrak {d}}_1(2\pi t))=\Delta _K^{-it} \end{aligned}$$

In particular, the translations are given by $U({\mathfrak {t}}(e^{2\pi t}-1))=\Delta _H^{-it}\Delta _K^{it}$, satisfy $U({\mathfrak {t}}(s))H\subset H$ for $s\ge 0$, $U({\mathfrak {t}}(1))H=K$ and have a positive generator.

Furthermore, the generator *P* of the translation group is $\frac{1}{2\pi }\left( \log (\Delta _K)- \log (\Delta _H) \right)$. In general, we have the relation $\log (\Delta _{U({\mathfrak {t}}(s))H}) = {{\mathrm{Ad\,}}}U({\mathfrak {t}}(s))(\log (\Delta _H)) = \log (\Delta _H) + 2\pi sP$.

#### Proof

We prove the last statement. The operator $\log (\Delta _K)- \log (\Delta _H)$ is essentially self-adjoint on its natural domain (one can prove this by first taking the Gårding domain). Thus, we can apply Trotter’s product formula:

$$\begin{aligned} e^{it(\log (\Delta _H)-\log (\Delta _K))}&={{\mathrm {s}\text {-}\lim }\,}_{n \rightarrow \infty }\left( \Delta _H^{i\frac{t}{n}}\Delta _K^{-i\frac{t}{n}} \right) ^n = {{\mathrm {s}\text {-}\lim }\,}_{n\rightarrow \infty }\left( U({\mathfrak {t}}(e^{-2\pi \frac{t}{n}}-1)) \right) ^n \\&={{\mathrm {s}\text {-}\lim }\,}_{n\rightarrow \infty }U\left( {\mathfrak {t}}(n(e^{-2\pi \frac{t}{n}}-1))\right) =U({\mathfrak {t}}(2\pi t)). \end{aligned}$$

The last relation follows from Lemma 2.1. $\square$

### The one-particle structure of the free scalar field

The relativistic invariance is encoded in the Poincaré group ${{\mathcal {P}}}$ on the $(D+1)$-dimensional Minkowski space time ${{\mathbb {R}}}^{D+1}$, where $D>1$. It is the semi-direct product of the full Lorentz group ${\mathcal {L}}$ and the translation group ${{\mathbb {R}}}^{D+1}$:

$$\begin{aligned} {{\mathcal {P}}}={\mathcal {L}}\ltimes {{\mathbb {R}}}^{D+1}. \end{aligned}$$

The subgroup ${{{\mathcal {P}}}^\uparrow _+}= {\mathcal {L}}^\uparrow _+\ltimes {{\mathbb {R}}}^{D+1}$ of time- and space orientation-preserving transformations gives the relativistic transformations from one inertial frame to another. The causal structure is determined by the Minkowski metric and the causal complement of a region $O\subset {{\mathbb {R}}}^{1+D}$ is determined as follows:

$$\begin{aligned} O'=\{x\in {{\mathbb {R}}}^{1+D}:(y-x)^2<0, y\in O\}^\circ . \end{aligned}$$

where $\circ$ denotes the open kernel.

We restrict our analysis to the scalar representations of ${{{\mathcal {P}}}^\uparrow _+}$ (see e.g. [Var85]). A **scalar representation with mass** $m \ge 0$ **(for** $D>1$**)** is defined on the Hilbert space ${\mathfrak {H}}_m= L^2(\Omega _m,d\Omega _m)$, where $d\Omega _m$ is the unique (up to a constant) Lorentz-invariant measure on the mass shell $\Omega _m=\{p=(p_0,\ldots ,p_D)\in {{\mathbb {R}}}^{D+1}: p^2=m^2, p_0\ge 0\}$. Let $\omega _m(\pmb {p})=\sqrt{m^2+|\pmb {p}|^2}$, then $\Omega _m=\{(\omega _m(\pmb {p}),\pmb {p}); \pmb {p}\in {{\mathbb {R}}}^{D}\}$ and the measure can be expressed (up to a constant) in the $\pmb {p}$-coordinates as:

3 $$\begin{aligned} d\Omega _m= \frac{d^{D}\pmb {p}}{\omega _m(\pmb {p})}. \end{aligned}$$

The action $U_m$ of $(\Lambda ,a)\in {{{\mathcal {P}}}^\uparrow _+}= {\mathcal {L}}^\uparrow _+\ltimes {{\mathbb {R}}}^{D+1}$ is given as follows:

4 $$\begin{aligned} (U_m((\Lambda ,a))\Psi )(p)=e^{ia\cdot p}\Psi (\Lambda ^{-1}p), \text { for } \Psi \in {\mathfrak {H}}_m. \end{aligned}$$

Consider the restriction *E* of the Fourier transformation on Schwartz functions on ${{\mathbb {R}}}^{D+1}$ to $\Omega _m$. Let ${{\mathcal {S}}}(\Omega _m)$ be the set of Schwartz functions on ${{\mathbb {R}}}^{D+1}$ restricted to $\Omega _m$, then

$$\begin{aligned} E:{{\mathcal {S}}}({{\mathbb {R}}}^{D+1})&\rightarrow {{\mathcal {S}}}(\Omega _m) \\ f&\mapsto (E f)(p)=\int _{{{\mathbb {R}}}^{D+1}} e^{ix\cdot p }f(x) d^{D+1}x, \ \ p \in \Omega _m \end{aligned}$$

(as $D> 1$, this holds even if $m=0$). Then $E({{\mathcal {S}}}({{\mathbb {R}}}^{D+1}))$ is dense in ${\mathfrak {H}}_m$. We refer to *p* as the momentum variable and *x* as the position variable. We shall denote by $\pmb {x}_\perp$ the $D-1$ coordinate vector $(x_2,\ldots ,x_D)$. The action of the Poincaré group on the one-particle space (4) is covariant with respect to the action of ${{{\mathcal {P}}}^\uparrow _+}$ on test functions in ${{\mathcal {S}}}({{\mathbb {R}}}^{D+1})$:

$$\begin{aligned} (U_m((\Lambda ,a))E f)(x) = E (f(\Lambda ^{-1}(x-a))). \end{aligned}$$

The local structure of the free scalar field is encoded in the local space corresponding to bounded open bounded regions $O \subset {{\mathbb {R}}}^{D+1}$:

5 $$\begin{aligned} H(O):= \overline{\{Ef \in L^2(\Omega _m,d\Omega _m): f\in {\mathcal {S}}({{\mathbb {R}}}^{D+1},{{\mathbb {R}}}), \mathrm{supp\,}(f)\subset O \}}. \end{aligned}$$

We shall call ${\mathcal {O}}$ the set of bounded, open regions. For an arbitrary regions *S* with non-empty interior in Minkowski space, its local subspace is generated by the subspaces of bounded open regions contained in it:

$$\begin{aligned} H(S)=\overline{\bigcup \limits _{O\subset S}H(O)}. \end{aligned}$$

The map ${{\mathcal {O}}}\ni O\mapsto H(O)\subset {{\mathcal {H}}}$ and the Poincaré representation $U_m$ define a net of standard subspaces, also called one-particle net or first quantized net, satisfying the following properties (see e.g. [BGL02]):

1. **Isotony:** $$H(O_1) \subset H(O_2)$$ for $$O_1 \subset O_2$$;
2. **Locality:** if $O_1\subset O_2'$, then $H(O_1)\subset H(O_2)'$, where $O'$ denotes the spacelike complement of *O*;
3. **Poincaré covariance:** $H(gO) = U(g)H(O)$ for $g \in {{{\mathcal {P}}}}^\uparrow _+$;
4. **Spectral condition:** the joint spectrum of the translation subgroup in *U* is contained in the closed forward light cone $\overline{V_+}=\{p\in {{\mathbb {R}}}^{D+1}: p^2\ge 0, p_0\ge 0\}$.
5. **Cyclicity**: *H*(*O*) are cyclic subspaces.

It is a consequence of locality and cyclicity that *H*(*O*) are standard subspaces if *O* has a nontrivial spacelike complement.

Consider the standard wedge $W_1=\{x\in {{\mathbb {R}}}^{D+1}:|x_0|<x_1\}$ in the $x_1$-direction and let

$$\begin{aligned} \Lambda _{W_1}(t)(x_0,x_1,\pmb {x}_\perp )=(\cosh (t)x_0+\sinh (t)x_1, \sinh (t)x_0+\cosh (t)x_1,\pmb {x}_\perp ) \end{aligned}$$

be the one-parameter group of Lorentz boosts fixing $W_1$. Any other region of the form $W=gW_1, g \in {{{\mathcal {P}}}^\uparrow _+}$ is also called a wedge, and we put $\Lambda _W(t) = g\Lambda _{W_1}(t)g^{-1}$ with such a *g* (this is well-defined, because any $g\in {{\mathcal {P}}}_+^\uparrow$ fixing *W* commutes with $\Lambda _W$). We shall denote by ${\mathcal {W}}$ the set of wedges. Let $H(W)=\overline{\bigcup _{O\subset W}H(O)}$ be the subspace associated to the wedge *W*. The net defined by (5) further satisfies the following properties.

(SS6) **Bisognano–Wichmann (BW) property:** Let $W\in {\mathcal {W}}$, it holds that
  $$\begin{aligned} U(\Lambda _{W}(t)) = \Delta _{H(W)}^{-\frac{it}{2\pi }}\qquad \text { for } t \in {{\mathbb {R}}}, \end{aligned}$$
(SS7) **Haag duality (for wedges):** $H(W')=H(W)'$, for all $W\in {\mathcal {W}}.$

### Second quantization and nets of von Neumann algebras

Let ${{\mathcal {H}}}$ be a Hilbert space and $H\subset {{\mathcal {H}}}$ a real linear subspace. The von Neumann algebra *R*(*H*), called second quantization algebras, on the symmetric Fock space ${\mathcal {F}}_+({{\mathcal {H}}})$ generated by the Weyl operators:

$$\begin{aligned} R(H) \equiv \{\mathrm {w}(\xi ): \xi \in H\}'', \end{aligned}$$

where $\mathrm {w}(\xi ), \xi \in {{\mathcal {H}}}$ are unitary operators on ${\mathcal {F}}_+({{\mathcal {H}}})$ characterized by

$$\begin{aligned} \mathrm {w}(\xi )e^\eta = e^{-\frac{1}{2}\langle \xi ,\xi \rangle -\langle \xi ,\eta \rangle }\cdot e^{\xi +\eta }, \end{aligned}$$

where $e^\eta = 1 \oplus \eta \oplus \frac{1}{\sqrt{2!}}\eta ^{\otimes 2} \cdots$ is a coherent vector in ${\mathcal {F}}_+({{\mathcal {H}}})$. By continuity in the strong operator topology of the map ${{\mathcal {H}}}\ni \xi \mapsto \mathrm {w}(\xi )\in {\mathcal {F}}_+({{\mathcal {H}}})$ we have that

$$\begin{aligned} R(H) = R(\overline{H})\ . \end{aligned}$$

Moreover, the Fock vacuum vector $\Omega = e^0$ is cyclic (respectively separating) for *R*(*H*) if and only if $\overline{H}$ is cyclic (respectively separating). Therefore one verifies the relation:

6 $$\begin{aligned} \langle \mathrm {w}(\xi )\Omega ,\mathrm {w}(\eta )\Omega \rangle =e^{-\frac{1}{2}(\left\Vert \xi \right\Vert ^2+\left\Vert \eta \right\Vert ^2)}e^{\langle \xi ,\eta \rangle }. \end{aligned}$$

The second quantization respects the lattice structure [Ara63] and the modular structure [LRT78, LMR16]. We shall denote $J_{R(H),\Omega }$, $\Delta _{R(H),\Omega }$ the Tomita operators associated with $(R_+(H),\Omega )$, and by $\Gamma _+(T)$ the multiplicative second quantization of a one-particle operator *T* on ${{\mathcal {H}}}$. It holds that $\Gamma _+(T)e^\xi =e^{T\xi }$ for $\xi \in {{\mathcal {H}}}$.

#### Proposition 2.4

[LRT78, LMR16] Let *H* and $H_\iota$ be closed, real linear subspaces of ${{\mathcal {H}}}$ with $\iota \in {\mathcal {J}}$ and ${\mathcal {J}}$ is an index set. We have

1. $J_{R(H),\Omega } = {\Gamma }_+(J_H)$, $\Delta _{R(H),\Omega }= {\Gamma }_+(\Delta _H)$ if *H* is standard;
2. $$R(H)' = R(H')$$;
3. $$R({\mathrm {Span}\,}_{\iota \in {\mathcal {J}}} H_\iota ) = \bigvee _{\iota \in {\mathcal {J}}} R(H_\iota )$$;
4. $$R(\bigcap _{\iota \in {\mathcal {J}}} H_\iota ) = \bigcap _{\iota \in {\mathcal {J}}} R(H_\iota )$$,

where $\bigvee _{\iota \in {\mathcal {J}}} R(H_\iota )$ denotes the von Neumann algebra generated by $R(H_\iota )$’s.

In particular, the second quantization promotes the one-particle net defined in (5) to a Haag–Kastler net of local algebras. Consider the map ${{\mathcal {O}}}\ni O\mapsto {{\mathcal {A}}}(O)=R(H(O))\subset {\mathcal {F}}_+({{\mathcal {H}}})$, together with the second quantization $U=\Gamma _+(U_m)$ of the one-particle Poincaré representation $U_m$, the following hold:

1. **Isotony:** $${{\mathcal {A}}}(O_1) \subset {{\mathcal {A}}}(O_2)$$ for $$O_1 \subset O_2$$;
2. **Locality:** if $O_1\subset O_2'$, then ${{\mathcal {A}}}(O_1)\subset {{\mathcal {A}}}(O_2)'$;
3. **Poincaré covariance:** $U(g){{\mathcal {A}}}(O)U(g)^*={{\mathcal {A}}}(gO)$ for $g\in {{\mathcal {P}}}_+^\uparrow$.
4. **Positivity of the energy:** the joint spectrum of the translation subgroup in *U* is contained in the closed forward light cone $\overline{V_+}$.
5. **Vacuum and the Reeh-Schlieder property:** $\Omega$ is the (up to a phase) unique vector such that $U(g)\Omega =\Omega$ for $g\in {{{\mathcal {P}}}^\uparrow _+}$ and is cyclic, $\overline{{{\mathcal {A}}}(O)\Omega }={{\mathcal {H}}}$ for any *O*.
6. **The Bisognano–Wichmann property:** For a wedge $W \in {\mathcal {W}}$, we put ${{\mathcal {A}}}(W)= \left( \bigvee _{O\subset W} {{\mathcal {A}}}(O)\right) ''$. Then it holds that
  $$\begin{aligned} U(\Lambda _{W}(t))=\Delta _{{{\mathcal {A}}}(W),\Omega }^{-\frac{it}{2\pi }}, \end{aligned}$$
  where $\Delta _{{{\mathcal {A}}}(W),\Omega }^{it}$ is the modular group of ${{\mathcal {A}}}(W)$ with respect to $\Omega$.

### The *U*(1)-current net

We introduce a family of standard subspaces parametrized by intervals on ${{\mathbb {R}}}$, the *U*(1)-current. Let us consider ${{\mathcal {H}}}_{\mathrm{U(1)}} = L^2({{\mathbb {R}}},d\theta ')$ and, for each $I \subset {{\mathbb {R}}}$ open interval, the subspace

$$\begin{aligned} H_\mathrm{U(1)}(I)=\overline{\{({{\hat{g}}}\circ {\mathfrak {e}})(\theta ') ;g\in C_0^\infty ({{\mathbb {R}}},{{\mathbb {R}}}), {{\hat{g}}}(0) = 0, \mathrm{supp\,}(g)\subset I\}} \subset L^2({{\mathbb {R}}},d\theta '), \end{aligned}$$

where ${{\hat{g}}}$ is the Fourier transform of *g* and $({{\hat{g}}}\circ {\mathfrak {e}})(\theta ') = {{\hat{g}}}(e^{-\theta '})$. Furthermore, we introduce

$$\begin{aligned} (U_{\mathrm{U(1)}}(\alpha , t)\xi )(\theta ') = e^{it e^{-\theta '}}\xi (\theta '-\alpha ). \end{aligned}$$

This is a unitary representation of the translation-dilation group ${\textbf {P}} = {{\mathbb {R}}}\ltimes {{\mathbb {R}}}$. Let $x\in {{\mathbb {R}}}$, then $(\alpha ,0)\in {{\mathbb {R}}}\ltimes {{\mathbb {R}}}$ corresponds to the dilation ${\mathfrak {d}}(\alpha ) x =e^{\alpha }x$ and $(0,t)\in {{\mathbb {R}}}\ltimes {{\mathbb {R}}}$ corresponds to the translations ${\mathfrak {t}}(t)x=x+t$. It is straightforward to check that $U_{\mathrm{U(1)}}(\alpha , t) H(I) = H(e^\alpha I + t)$.

Each *H*(*I*) is a standard subspace and for disjoint $I_1, I_2$ it holds that $H(I_1) \subset H(I_2)'$. Furthermore, the Bisognano–Wichmann property holds: $\Delta _{H({{\mathbb {R}}}^+)}^{it}=U_{\mathrm{U(1)}}(-2\pi t,0), t\in {{\mathbb {R}}}$.

Let $f,g \in C_0^\infty ({{\mathbb {R}}},{{\mathbb {R}}})$ with ${\hat{f}}(0)={\hat{g}}(0)=0$ and call the primitives *F*, *G*, respectively. The condition ${\hat{f}}(0)={\hat{g}}(0)=0$ implies $F,G \in C_0^\infty ({{\mathbb {R}}},{{\mathbb {R}}})$. We have, under the substitution $p=e^{-\theta ^\prime }$, that

$$\begin{aligned} \langle {\hat{g}}\circ {\mathfrak {e}},{\hat{f}}\circ {\mathfrak {e}} \rangle&=\int _{{{\mathbb {R}}}_+}{\hat{g}}(-p){\hat{f}}(p)\frac{dp}{p} =\int _{{{\mathbb {R}}}_+}{\hat{G}}(-p){\hat{F}}(p)pdp. \end{aligned}$$

where $\langle {\hat{g}}\circ {\mathfrak {e}},{\hat{f}}\circ {\mathfrak {e}} \rangle$ has to be intended as the Lebesgue scalar product on ${{\mathbb {R}}}$ of the functions ${{\mathbb {R}}}\ni \theta '\mapsto ({{\hat{g}}}\circ {\mathfrak {e}})(\theta ')$ and ${{\mathbb {R}}}\ni \theta '\mapsto ({{\hat{f}}}\circ {\mathfrak {e}})(\theta ')$. The imaginary part of the scalar product (symplectic form) plays a crucial role in the second quantization

7 $$\begin{aligned} \mathrm {Im}\,\langle {\hat{g}}\circ {\mathfrak {e}},{\hat{f}}\circ {\mathfrak {e}} \rangle&=\frac{1}{2i}\int _{{{\mathbb {R}}}_+}\left( {\hat{G}}(-p){\hat{F}} (p)-{\hat{F}}(-p){\hat{G}}(p)\right) pdp=\frac{1}{2}\int _{{{\mathbb {R}}}} G(x)f(x)dx \end{aligned}$$

The family $\{H(I)\}$ and the representation $U_{\mathrm{U(1)}}$ are unitarily equivalent to the family of closed real subspaces coming from the $\mathrm{U(1)}$-current conformal net and the one-particle symmetry restricted to the translation-dilation group, see [BT15,  Section 5.2]. The intertwining map for $h\in C_0^\infty ({{\mathbb {R}}},{{\mathbb {R}}})$ is ${\hat{h}}(p)\mapsto \widehat{h^\prime }(e^{-\theta '})$. Thus to switch to the standard definition of the *U*(1)-current presented in the literature (see e.g. [Lon08]) one replaces $g\in C_0^\infty ({{\mathbb {R}}},{{\mathbb {R}}})$ with ${\hat{g}}(0)=0$ with its primitive $G\in C_0^\infty ({{\mathbb {R}}},{{\mathbb {R}}})$.

## Free Scalar Field on the Null Plane

### Direct integrals and decompositions

Let us summarize some of the basic notions and results on the direct integral of Hilbert spaces and decomposition of group representations. We follow the conventions of [Dix81], see also [KR97].

Let *X* be a $\sigma$-compact locally compact Borel measure space, $\nu$ the completion of a Borel measure on *X* and $\{{{\mathcal {K}}}_\lambda \}$ a family of separable Hilbert spaces indexed by $\lambda \in X$. We say that a separable Hilbert space ${{\mathcal {K}}}$ is the **direct integral** of $\{ {{\mathcal {K}}}_\lambda \}$ over $(X,\nu )$ if, to each $\xi \in {{\mathcal {K}}}$ there corresponds a function $\lambda \mapsto \xi (\lambda ) \in {{\mathcal {K}}}_\lambda$ and

1. $\lambda \mapsto \langle \xi (\lambda ),\eta (\lambda ) \rangle$ is $\nu$-integrable and $\langle \xi ,\eta \rangle =\int _X \langle \xi (\lambda ),\eta (\lambda ) \rangle d\nu (\lambda )$
2. if $\phi _\lambda \in {{\mathcal {K}}}_\lambda$ for all $\lambda$ and $\lambda \mapsto \langle \phi _\lambda ,\eta (\lambda ) \rangle$ is integrable for all $\eta \in {{\mathcal {K}}}$, there is $\phi \in {{\mathcal {K}}}$ such that $\phi (\lambda )=\phi _\lambda$ for almost every $\lambda$.

In this case, we write:

$$\begin{aligned} {{\mathcal {K}}}=\int _X^\oplus {{\mathcal {K}}}_\lambda d\nu (\lambda ). \end{aligned}$$

An operator $T\in {{\mathcal {B}}}({{\mathcal {K}}})$ is said to be **decomposable** when there is a function $\lambda \mapsto T_\lambda$ on *X* such that $T_\lambda \in {{\mathcal {B}}}({{\mathcal {K}}}_\lambda )$ and, for each $\xi \in {{\mathcal {K}}}$, $T_\lambda \xi (\lambda )=(T\xi )(\lambda )$ for $\nu$-almost every $\lambda$, and in this case we write $T = \int _X^\oplus T_\lambda d\nu (\lambda )$. If $T_\lambda =f(\lambda )1$ for some $f\in L^\infty (X,\nu )$, we say that *T* is **diagonalizable**. An operator $T\in {{\mathcal {B}}}({{\mathcal {K}}})$ is decomposable if and only if *T* commutes with every diagonalizable operator. Conversely, let $X\ni \lambda \rightarrow T_\lambda \in {{\mathcal {B}}}({{\mathcal {K}}}_\lambda )$ be a field of bounded operators with $\sup _\lambda \Vert T_\lambda \Vert < \infty$. If for any $\xi \in {{\mathcal {K}}}$ with $\xi =\int _X^\oplus \xi (\lambda ) d\nu (\lambda )$ there exists $\eta \in {{\mathcal {K}}}$ such that $\eta (\lambda )=T_\lambda \xi (\lambda )$ for almost every $\lambda \in X$, then $T=\int _X^\oplus T_\lambda d\nu (\lambda )\in {{\mathcal {B}}}({{\mathcal {K}}})$ defines a bounded decomposable operator on ${{\mathcal {K}}}$ and $\lambda \rightarrow T_\lambda$ is called a *measurable field of bounded operators*. We shall use the notation ${{\mathcal {K}}}=\int _X^{\oplus _{{\mathbb {R}}}} {{\mathcal {K}}}_\lambda d\nu (\lambda )$ when we consider a direct integral of real Hilbert spaces.

The following Lemma can be found in [MT18,  Appendix B].

#### Lemma 3.1

Let $${{\mathcal {K}}}=\int _X^\oplus {{\mathcal {K}}}_\lambda d\nu (\lambda )$$ then

- Let $H=\int _X^{\oplus _{{\mathbb {R}}}} H_\lambda d\nu (\lambda )\subset {{\mathcal {K}}}$ be a real subspace such that $H_\lambda \subset {{\mathcal {K}}}_\lambda$ is a real subspace, then $H'=\int _X^{\oplus _{{\mathbb {R}}}} H'_\lambda d\nu (\lambda )$.
- Let $\{H_k\}_{k\in {{\mathbb {N}}}}$ be a countable family of real subspaces of ${{\mathcal {K}}}$ such that $H_k=\int _X^{\oplus _{{\mathbb {R}}}} (H_k)_\lambda d\nu (\lambda )$ on ${{\mathbb {R}}}$ and $(H_k)_\lambda \subset {{\mathcal {K}}}_\lambda$ is a real subspace, then $\bigcap _{k\in {{\mathbb {N}}}} H_k=\int _X^{\oplus _{{\mathbb {R}}}} \bigcap _{k\in {{\mathbb {N}}}} (H_k)_\lambda d\nu (\lambda )$.
- Let $\{H_k\}_{k\in {{\mathbb {N}}}}$ be a countable family of real subspaces of ${{\mathcal {K}}}$ such that $H_k=\int _X^{\oplus _{{\mathbb {R}}}} (H_k)_\lambda d\nu (\lambda )$ on ${{\mathbb {R}}}$ and $(H_k)_\lambda \subset {{\mathcal {K}}}_\lambda$ is a real subspace, then $\overline{{\mathrm {Span}\,}_{k\in {{\mathbb {N}}}} H_k}=\int _X^{\oplus _{{\mathbb {R}}}} \overline{{\mathrm {Span}\,}_{k\in {{\mathbb {N}}}} (H_k) }_\lambda d\nu (\lambda )$.

It follows immediately that the direct integral of standard subspaces is again a standard subspaces on the direct integral of Hilbert space.

Let *G* be a locally compact group and $\pi$ a continuous (in the strong operator topology) unitary representation of *G* on ${{\mathcal {K}}}=\int ^\oplus _X {{\mathcal {K}}}_\lambda d\nu (\lambda )$. Suppose that, for each $g\in G$, we have $\pi (g)=\int ^\oplus \pi _\lambda (g) d\mu (\lambda )$, then we say that $\pi$ is the **direct integral** of the $\pi _\lambda$ and write:

$$\begin{aligned} \pi =\int ^\oplus _X \pi _\lambda d\nu (\lambda ). \end{aligned}$$

Equivalently, $\pi$ is a direct integral if each $\pi (g), \ g\in G$, is decomposable. As a consequence a direct integral of Poincaré covariant nets of standard subspaces is a Poincaré covariant net of standard subspaces on the direct integral of Hilbert space.

As a particular case, let ${\mathcal {K}}= \int _X^\oplus {\mathcal {K}}_0 d\nu$ be a direct integral Hilbert space over the constant field ${\mathcal {K}}_0$, which is a separable Hilbert space, on *X* with measure $\nu$. Then it holds that [Dix81,  Proposition II.1.8.11, Corollary]

8 $$\begin{aligned} \int _X^\oplus {\mathcal {K}}_0 d\nu (\lambda ) \simeq L^2(X,\nu )\otimes {\mathcal {K}}_0. \end{aligned}$$

The isomorphism is given by identifying $L^2(X,\nu )\otimes {{\mathcal {K}}}_0$ as the space of ${{\mathcal {K}}}_0$-valued $L^2$-functions. By this isomorphism, we identify $\int _X^\oplus T d\nu (\lambda )$ and ${\mathbbm {1}}\otimes T$, where $T\in {\mathcal {B}}({\mathcal {K}}_0)$ (a constant field of bounded operators).

### Decomposition of the one-particle space

Let us fix $D>1$. We first consider the $m>0$ case. In Remark 3.5 we explain the minor modification to deal with the massless case.

The hypersurface $X_-^0 := \{x\in {{\mathbb {R}}}^{D+1}:x_0-x_1=0\}$ is called the **null plane** in the $x_1$-direction. It is appropriate to consider the coordinate frame $(x_+,x_-,\pmb {x}_\perp )=(\frac{x_0+x_1}{\sqrt{2}}, \frac{x_0-x_1}{\sqrt{2}}, \pmb {x}_\perp )$. In these coordinates, the Minkoswki product becomes

9 $$\begin{aligned} x\cdot p= x_+p_-+x_-p_+-\pmb {x}_\perp \pmb {p}_\perp \end{aligned}$$

and the Minkowski (pseudo)norm $x^2=2x_+x_--\pmb {x}_\perp ^2$. In the momentum space, the massive hyperboloid is determined by $2p_+p_- -\pmb {p}_\perp ^2= m^2$, and for each $(p_-, \pmb {p}_\perp ) \in {{\mathbb {R}}}_+ \times {{\mathbb {R}}}^{D-1}$ there is one and only one $p_+ \in {{\mathbb {R}}}_+$ satisfying this equation. For a test function $f=f(x_+,x_-,\pmb {x}_\perp )$ on ${{\mathbb {R}}}^{D+1}$, let *Ef* be its Fourier transform restricted to the mass hyperboloid $\Omega _m= \left\{ p=\big ({\frac{m^2+\pmb {p}_\perp ^2}{2p_-}}, p_-,\pmb {p}_\perp \big ): p_->0, {\varvec{p}}_\perp \in {{\mathbb {R}}}^{D-1}\right\}$ (in the $(p_+,p_-,\pmb {p}_\perp )$-coordinates). In the $(p_-,\pmb {p}_\perp )$-coordinates, we have, up to a unitary (given by the change of variables),

$$\begin{aligned} {\mathfrak {H}}_m\simeq L^2\left( {{\mathbb {R}}}^D, \frac{d^{D}\pmb {p}}{\omega _m(\pmb {p})}\right) \simeq L^2\left( {{\mathbb {R}}}_+ \times {{\mathbb {R}}}^{D-1}, \frac{2p_-dp_-d\pmb {p}_\perp }{m^2+2p_-^2+\pmb {p}_\perp ^2}\right) \end{aligned}$$

(since $dp_1d\pmb {p}_\perp =\frac{1}{\sqrt{2}}dp_-\pmb {p}_\perp$ and $\omega _m (p_-,\pmb {p}_\perp )=\frac{1}{\sqrt{2}}\left( \frac{m^2+\pmb {p}_\perp ^2}{2p_-}+p_-\right)$ is the dispersion relation in the $(p_-,\pmb {p}_\perp )$-coordinates). For sake of notational simplicity, we will write $\xi (p_1,\pmb {p}_\perp )\in {\mathfrak {H}}_m$ or $\xi (p_-,\pmb {p}_\perp ) \in {\mathfrak {H}}_m$ for $\xi \in {\mathfrak {H}}_m$ in terms of $(p_1,\pmb {p}_\perp )$ or $(p_-,\pmb {p}_\perp )$, respectively. In the same way, the representation $U_m$ acts on ${\mathfrak {H}}_m$ in various realizations.

We introduce the map $V_{\mathrm M}:{\mathfrak {H}}_m= L^2(\Omega _m,d\Omega _m) \rightarrow L^2({{\mathbb {R}}}^{D},d\theta 'd^{D-1}\pmb {p}_\perp )$ as follows: Let $\xi \in L^2({{\mathbb {R}}}^D,d\theta 'd^{D-1}\pmb {p}_\perp )$,

$$\begin{aligned} (V_{\mathrm M}^{-1}\xi )(p_1,\pmb {p}_\perp )&= \textstyle {\xi \left( \mathrm {\mathrm {arcsinh}}\left( \frac{p_1}{\omega _m(\pmb {p}_\perp )}\right) \right) -\log (\omega _m(\pmb {p}_\perp ))+\log \sqrt{2}, \pmb {p}_\perp )}, \end{aligned}$$

where $\omega _m(\pmb {p}_\perp ) := \sqrt{m^2 + \pmb {p}_\perp ^2}$. $V_\mathrm {M}$ is a unitary operator. Indeed, first, the change of $p_1$ to rapidity $\theta =\mathrm {\mathrm {arcsinh}}(\frac{p_1}{\omega _m(\pmb {p}_\perp )})$ for a fixed $\pmb {p}_\perp$, or $p_1 = \omega _m(\pmb {p}_\perp )\sinh \theta$ is a smooth one-to-one map ${{\mathbb {R}}}^D\rightarrow {{\mathbb {R}}}^D$. Moreover, with $\frac{\partial \theta }{\partial p_1} = \frac{1}{\omega _m(\pmb {p}_\perp )}/\sqrt{1+\frac{p_1^2}{\omega _m(\pmb {p}_\perp )^2}} = \frac{1}{\omega _m(\pmb {p})}$, the measure $d\Omega _m$ transforms in the following way, cf. (3):

$$\begin{aligned} d\Omega _m&=\frac{d^{D}\pmb {p}}{\omega _m(\pmb {p})}=d\theta d^{D-1}\pmb {p}_\perp . \end{aligned}$$

Therefore, the pullback of the rapidity substitution imposes the equivalence:

$$\begin{aligned} {\mathfrak {H}}_m=L^2(\Omega _m,d\Omega _m)\simeq L^2({{\mathbb {R}}}\times {{\mathbb {R}}}^{D-1},d\theta \times d^{D-1}\pmb {p}_\perp ) (\simeq L^2({{\mathbb {R}}},d\theta )\otimes L^2({{\mathbb {R}}}^{D-1},d^{D-1}\pmb {p}_\perp )). \end{aligned}$$

Moreover, the substitution $\theta '=\theta - \log (\omega _m(\pmb {p}_\perp ))+\log \sqrt{2}$ is a smooth one-to-one map: ${{\mathbb {R}}}\rightarrow {{\mathbb {R}}}$ if $m>0$ with $d\theta =d\theta '$.

By substituting $\theta = \mathrm {arcsinh}(\frac{p_1}{\omega _m(\pmb {p}_\perp )})$ or $p_1 = \omega _m(\pmb {p}_\perp )\sinh \theta$ in $p_- = \frac{1}{\sqrt{2}}(\omega _m(p_1,\pmb {p}_\perp ) - p_1)$ we have $p_-= \frac{1}{\sqrt{2}}\omega _m(\pmb {p}_\perp )e^{-\theta }$ therefore,

$$\begin{aligned} p_-= \frac{1}{\sqrt{2}}\omega _m(\pmb {p}_\perp )e^{-\theta '-\log (\omega _m(\pmb {p}_\perp ))+\log \sqrt{2}}=e^{-\theta '}, \end{aligned}$$

and

$$\begin{aligned} (V_{\mathrm M} \xi )(\theta ',\pmb {p}_\perp )=\xi (e^{-\theta '}, \pmb {p}_\perp ), \quad \xi \in L^2\left( {{\mathbb {R}}}_+ \times {{\mathbb {R}}}^{D-1}, \frac{2p_-dp_-d\pmb {p}_\perp }{m^2+2p_-^2+\pmb {p}_\perp ^2}\right) \end{aligned}$$

#### Proposition 3.2

We have the following equivalence, where the map is given by $V_{\mathrm M}$ composed by the inverse Fourier transform on the perpendicular momenta:

10 $$\begin{aligned} {\mathfrak {H}}_m\simeq L^2({{\mathbb {R}}}^{D},d\theta 'd^{D-1}\pmb {x}_\perp ) \simeq \int _{{{\mathbb {R}}}^{D-1}}^\oplus L^2({{\mathbb {R}}},d\theta ')d^{D-1}\pmb {x}_\perp , \end{aligned}$$

where the last equivalence is given by (8).

Subsequently, we will refer to these direct integral representations of ${\mathfrak {H}}_m$ as **spatial decomposition** and denote the intertwining unitary by $V_{\mathrm S}$, while the map $V_{\mathrm M}$ is referred to as **momentum decomposition**.

Next we discuss the decomposition of the spacetime symmetry. Let us take the wedge $W_1$ in the $x_1$-direction and consider the subgroup $\Lambda _{1}\ltimes {\mathfrak {t}}_{x_+}\subset {{{\mathcal {P}}}^\uparrow _+}$ consisting of boosts $\Lambda _{1}:=\Lambda _{W_1}$ along the $x_1$-direction and lightlike translations ${\mathfrak {t}}_{x_+}$ of $x_+$. It is straightforward that this group is isomorphic to the translation-dilation group ${\mathbf {P}}$ of ${{\mathbb {R}}}$. We will show that the restriction of $U_m$ to ${\mathbf {P}}$ decomposes with respect to the decompositions (10) of the one-particle vectors.

#### Lemma 3.3

The unitary $V_{\mathrm M}$ between ${\mathfrak {H}}_m$ and $\int _{{{\mathbb {R}}}^{D-1}}^\oplus L^2({{\mathbb {R}}},d\theta ') d^{D-1}\pmb {p}_\perp$ intertwines the actions of the subgroup ${\mathbf {P}}$ to:

11 $$\begin{aligned} (V_{\mathrm M} U_m(\Lambda _{1}(\alpha ),{\mathfrak {t}}_{x_+}(a_+))\xi )(\theta ',\pmb {p}_\perp )&= e^{i a_+e^{-\theta '} }(V_{\mathrm M}\xi )(\theta '-\alpha ,\pmb {p}_\perp ), \end{aligned}$$

#### Proof

We start with the Poincaré group action given by $U_m$ on ${\mathfrak {H}}_m$ expressed in (4). We recall that $p_-= e^{-\theta '}$. Then the result is immediate by considering (9) and the fact that $a_+\cdot p_- =a_+e^{-\theta '}$,

For boosts, the claim follows directly from the following

$$\begin{aligned} \Lambda _{1}^{-1}(\alpha )p&=\left( \begin{array}{c} \cosh (\alpha )p_0 - \sinh (\alpha )p_1 \\ -\sinh (\alpha )p_0+\cosh (\alpha )p_1\\ \pmb {p}_\perp \end{array} \right) \\&= \left( \begin{array}{c} \omega _m(\pmb {p}_\perp ) \cosh (\theta '+\log (\omega _m(\pmb {p}_\perp )) - \log \sqrt{2}- \alpha ) \\ \omega _m(\pmb {p}_\perp )\sinh (\theta '+\log (\omega _m(\pmb {p}_\perp ))- \log \sqrt{2}-\alpha )\\ \pmb {p}_\perp \end{array} \right) . \end{aligned}$$

$$\square $$

#### Proposition 3.4

Under the spatial decomposition, the representation of $U_m$ to ${\mathbf {P}}$ is decomposable (in the sense of Sect. 3.1) and acts as follows:

12 $$\begin{aligned} (U_m((\Lambda _{1}(\alpha ),{\mathfrak {t}}_{x_+}(a_+)))\xi )(\theta ',\pmb {x}_\perp )&= e^{ia_+e^{-\theta '}}\xi (\theta '-\alpha , \pmb {x}_\perp ). \end{aligned}$$

#### Proof

The representation $U_m$ decomposes in the momentum decomposition as (11), and the action of $U_m$ does not involve the variable $\pmb {p}_\perp$, it commutes with the inverse Fourier transform on $\pmb {p}_\perp$. $\square$

#### Remark 3.5

The disintegration (10) and the identifications (11) and (12) apply also to the $m=0$ case up to a measure zero set. To see this, note that in the massless case $\Omega _0=\partial V_+=\{p\in {{\mathbb {R}}}^{D+1}: p^2=0,p_0>0\}$. The change of variables determining $V_{\mathrm M}^{-1}$ (both in $(p_1,\pmb {p}_\perp )$ or $(p_-,\pmb {p}_\perp )$ set of coordinates) are 1-1 diffeomorphisms on the set $\Omega _0\cap \{p\in {{\mathbb {R}}}^{D+1}: \pmb {p}_\perp \ne 0\}$. This set is dense in $\Omega _0$ with respect to the topology induced by the euclidean topology on ${{\mathbb {R}}}^{D+1}$. The complement $N_0=\{p\in \Omega _0:\pmb {p}_\perp = 0\}$ has measure zero with respect to the Lorentz invariant measure on $\Omega _0$, see (3). We conclude that $V_{\mathrm M}$ is a unitary operator. As $\Lambda _{1}$-boosts fix $N_0$, (10), (11) and (12) continue to hold (up to a measure zero set). This is the only modification to have in mind along the paper to adapt the massless case.

**Fig. 1:**
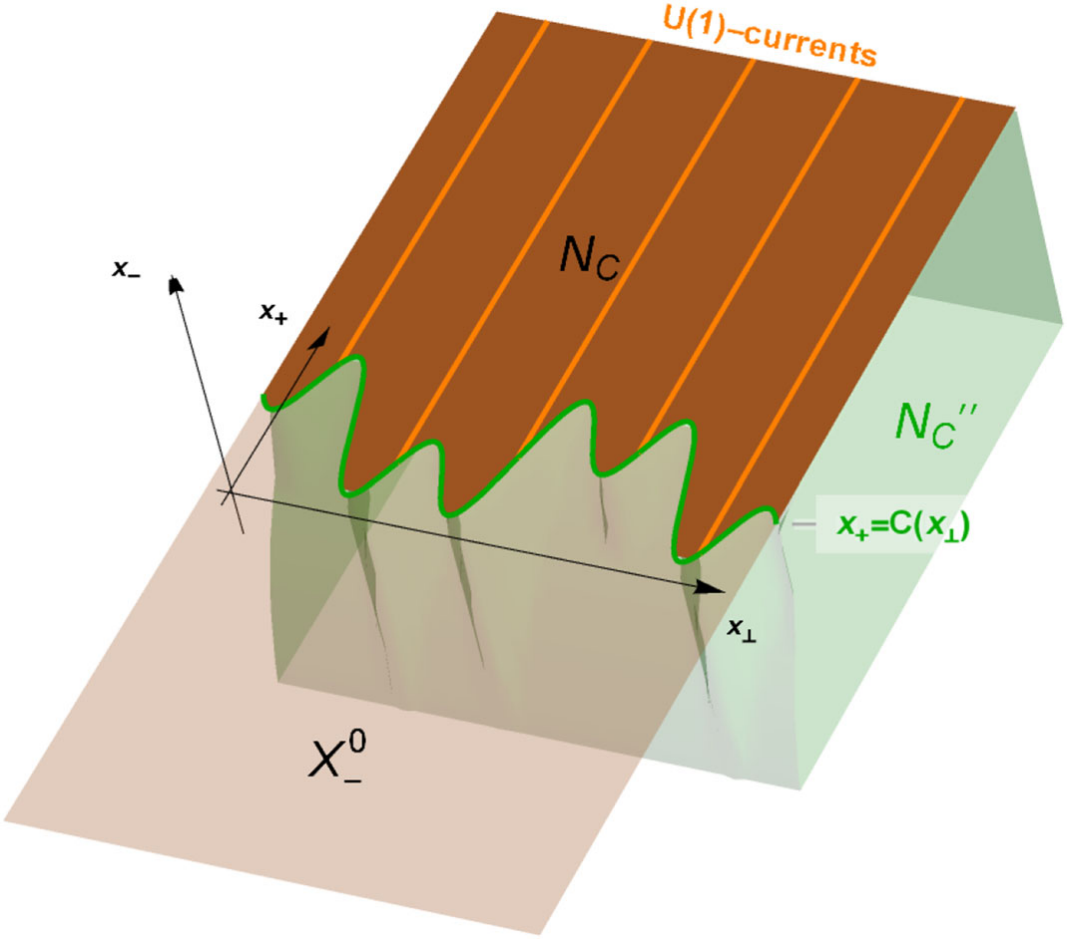
The geometric setup and illustration of the decomposition of standard subspaces of null cuts

### The local subspace of the null plane

Here we show that the free field can be restricted to regions on the null plane if we exclude the lightlike zero-modes (see Lemma 3.6 for the precise restriction, cf. [Ull04]). Recall that the algebra ${{\mathcal {A}}}(O)$ is generated by the Weyl operators associated with test functions supported in *O*. In this spirit, we define the local subspaces of open (with the relative topology) regions *R* in the null plane $X_-^0:=\{x\in {{\mathbb {R}}}^{D+1};x_-=0\}$. The geometric setup is visualised in Fig. 1. To make it precise, we consider distributions of the form

13 $$\begin{aligned} g_0(x_+,x_-,\pmb {x}_\perp )=\delta (x_-)g(x_+,\pmb {x}_\perp ), \end{aligned}$$

where *g* is a real-valued function such that ${{\mathbb {R}}}\times {{\mathbb {R}}}^{D-1}\ni ( x_+,\pmb {x}_\perp )\mapsto g(x_+,\pmb {x}_\perp )$ is a compactly supported smooth function. We denote the set of such distributions by ${\mathscr {D}}(X_-^0,{{\mathbb {R}}})$, and call them **thin test functions** supported on $X_-^0$.

The Fourier transform of such $g_0$ is a smooth function and can be restricted to the mass shell, which we denote by $Eg_0$. Then it is natural to consider one-particle vectors associated with thin test functions.

#### Lemma 3.6

Let $g_0(x_+,x_-,\pmb {x}_\perp )= \delta (x_-)g(x_+, \pmb {x}_\perp )$ as in (13). Then $E g_0 \in L^2(\Omega _m, d\Omega _m)$ (the norm is finite) if and only if $\int dx_+ g(x_+, \pmb {x}_\perp ) = 0$ for each $\pmb {x}_\perp$.

#### Proof

We take $g_0(x_+,x_-,\pmb {x}_\perp )= \delta (x_-)g(x_+, \pmb {x}_\perp )$ as above. The Fourier transform of $g_0$ is

$$\begin{aligned} (E g_0)(p)&=\int _{{{\mathbb {R}}}^{D+1}} e^{i(x_+p_-+x_-p_+-\pmb {x}_\perp \cdot \pmb {p}_\perp )}\delta (x_-)g(x_+, \pmb {x}_\perp )dx_+dx_-d^{D-1}\pmb {x}_\perp ={{\hat{g}}}(p_-, \pmb {p}_\perp ). \end{aligned}$$

By rapidity substitution $p_- =\frac{1}{\sqrt{2}}\omega _m(\pmb {p}_\perp )e^{-\theta }$ and $p_+=\frac{1}{\sqrt{2}}\omega _m(\pmb {p}_\perp )e^{\theta }$ we have:

$$\begin{aligned} \left\Vert E g_0\right\Vert ^2_{{\mathfrak {H}}_m^-}&=\int _{{{\mathbb {R}}}\times {{\mathbb {R}}}^{D-1}} \left| {{\hat{g}}}\left( \frac{1}{\sqrt{2}}\omega _m(\pmb {p}_\perp )e^{-\theta }, \pmb {p}_\perp \right) \right| ^2d\theta d^{D-1}\pmb {p}_\perp \\&=\int _{{{\mathbb {R}}}\times {{\mathbb {R}}}^{D-1}} |{{\hat{g}}}(e^{-\theta '}, \pmb {p}_\perp )|^2d\theta ' d^{D-1}\pmb {p}_\perp \end{aligned}$$

as we did in (10). Therefore, it is necessary that ${\hat{g}}(0,\pmb {p}_\perp ) = 0$ for each $\pmb {p}_\perp$ in order to have $E g_0\in {\mathfrak {H}}_m$ (that is the integral is finite), and equivalently, $\int dx_+ g(x_+, \pmb {x}_\perp ) = 0$ for each $\pmb {x}_\perp$.

Conversely, if $\int dx_+ g(x_+, \pmb {x}_\perp ) = 0$ for each $\pmb {x}_\perp$, then ${{\hat{g}}}(0, \pmb {p}_\perp )= 0$. In particular, since *g* is smooth, by the Taylor formula, $\theta '\mapsto \hat{g}(e^{-\theta '},\pmb {p}_\perp )$ has finite $L^2$-norm at $+\infty$. Then, since *g* is compactly supported, it decays fast when $p_-\rightarrow +\infty (\theta ^{\prime }\rightarrow -\infty )$ and $|\pmb {p}_\perp |\rightarrow +\infty$. As a consequence $E g_0\in {\mathfrak {H}}_m$. $\square$

The condition $\int dx_+ g(x_+, \pmb {x}_\perp ) = 0$ for each $\pmb {x}_\perp$ is satisfied if and only if *g* is the partial derivative in $x_+$ of another compactly supported smooth function. We define, for an open region *R* on the null plane $X^0_-$, as follows:

$$\begin{aligned} H(R) :={\overline{{\mathrm {Span}\,}}}^{{\mathfrak {H}}_m}{\{E g_0 \in L^2(\Omega _m,d\Omega _m):g_0=\delta (x_-)\partial _{x_+}g(x_+,\pmb {x}_\perp )\in {\mathscr {D}}(X_-^0,{{\mathbb {R}}}), \mathrm{supp\,}g \subset R\}}. \end{aligned}$$

In particular, a **null cut** is a region on the null plane associated with a continuous function $C:{{\mathbb {R}}}^{D-1}\rightarrow {{\mathbb {R}}}$, where ${{\mathbb {R}}}^{D-1}$ denotes the subspace $\{\pmb {x}\in {{\mathbb {R}}}^{D+1};x_-=x_+=0\}$

$$\begin{aligned} N_C:=\{\pmb {x}=(x_+,x_-,\pmb {x}_\perp ) \in {{\mathbb {R}}}^{D+1};x_-=0,x_+>C(\pmb {x}_\perp ) \}. \end{aligned}$$

These subspaces are natural in the view of the following isotony in an extended sense.

#### Lemma 3.7

Let $R\subset X_-^0$ be open (relatively in $X_-^0$) and $O\subset {{\mathbb {R}}}^{D+1}$ open such that there is a direction $a \in {{\mathbb {R}}}^{D+1}$ where $R\subset O + ta$ for every $t\in (0,\epsilon )$ for sufficiently small $\epsilon$. Then it holds that $H(R)\subset H(O)$.

#### Proof

Let *g* be any smooth function compactly supported in *R*. Then for $g_-$ a test function on *R* such that $g_-(t) \ge 0$ and $\int g_-(t)dt = 1$, $g(x_-, x_+, \pmb {x}_\perp ) = g_-(x_-)\partial _{x_+}g(x_+, \pmb {x}_\perp )$ has a Fourier transform in ${\mathfrak {H}}_m= L^2(\Omega _m,d\Omega _m)$. Indeed, we know that the Fourier transform of $\partial _{x_+}g(x_+, \pmb {x}_\perp )$ alone is in ${\mathfrak {H}}_m$, and it gets multiplied by ${{\hat{g}}}_-(p_+)$, which is rapidly decreasing. If we consider the scaled function $g_{-,n}(x_-) = ng_-(nx_-)$, its Fourier transform is ${{\hat{g}}}_-(\frac{p_+}{n})$, which is bounded and converges to 1, therefore, $E(g_{-,n}g)$ converges to $Eg_0$.

On the other hand, for fixed *a* and *t* as in the statement, for sufficiently large *n*, $g_{-,n}g$ is supported in $O + ta$, hence we have $Eg_0 \subset H(O+ta)$ by the closedness of $H(O+ta)$. This implies $H(R) \subset H(O+ta) = U_m(ta,0)H(O)$, and by the continuity of $U_m$, we obtain $H(R) \subset H(O)$. $\square$

The spaces *H*(*R*) are real subspaces of ${\mathfrak {H}}_m$. Recalling the spatial decomposition (10) of ${\mathfrak {H}}_m$, we will show that the same decomposition holds as a real Hilbert space through $V_{\mathrm S}$.

Let $I \subset {{\mathbb {R}}}$ be an open interval and $I_\perp \subset {{\mathbb {R}}}^{D-1}$ a open region. Under the spatial decomposition (10), we consider the local subspace $H(I\times I_\perp )$ of rectangular regions as follows

$$\begin{aligned} I\times I_\perp = \{(x_+,x_-,\pmb {x}_\perp ): x_-=0, x_+ \in I, \pmb {x}_\perp \in I_\perp \}. \end{aligned}$$

#### Proposition 3.8

Let $I\times I_\perp$ be a rectangle on the null plane $X_-^0$. Under the spatial decomposition (10), the local subspace $H(I\times I_\perp )$ decomposes in the following way (see e.g. [MT18,  Proposition B.2]):

14 $$\begin{aligned} H(I\times I_\perp )&\rightarrow \int ^{\oplus _{{\mathbb {R}}}}_{I_\perp } H_{U(1)}(I) d^{D-1}\pmb {x}_\perp \\ Eg_0&\mapsto V_{\mathrm S}Eg_0,\nonumber \end{aligned}$$

where $g_0(x_-,x_+,\pmb {x}_\perp )= \delta (x_-)g(x_+, \pmb {x}_\perp )\in {\mathscr {D}}(X_-^0,{{\mathbb {R}}})$ satisfying $\int dx_+g(x_+, \pmb {x}_\perp )=0$ for each $\pmb {x}_\perp \in I_\perp$.

*H*(*R*) is separating for any $R = I\times I_\perp$ where $I\subsetneq {{\mathbb {R}}}$, and standard if *I* is not empty and $I_\perp = {{\mathbb {R}}}^{D-1}$.

#### Proof

By the spatial decomposition (10), we identify the whole Hilbert space ${\mathfrak {H}}_m$ with $\int _{{{\mathbb {R}}}^{D-1}}^\oplus L^2({{\mathbb {R}}},d\theta ')d\pmb {x}_\perp$. By (8) the right-hand side of (14) is the constant field of standard spaces $\int _{I_\perp }^{\oplus _{{\mathbb {R}}}} H_{U(1)}(I)d\pmb {x}_\perp$ of real Hilbert spaces and is identified with $H_{U(1)}(I)\otimes _{{\mathbb {R}}}L^2(I_\perp ,{{\mathbb {R}}}, d\pmb {x}_\perp )$. On the other hand, $H( I\times I_\perp )$ is generated by the functions of the form ${{\hat{g}}}_+(e^{-\theta '})g_\perp (\pmb {x}_\perp )$, where $\mathrm{supp\,}g_+ \Subset I, {{\hat{g}}}_+(0) = 0$ and $\mathrm{supp\,}g_\perp \Subset I_\perp$, hence, by Lemma 3.6, $H( I\times I_\perp )=\int ^{\oplus _{{\mathbb {R}}}}_{I_\perp } H_{U(1)}(I) d^{D-1}\pmb {x}_\perp$ in the direct integral disintegration.

The statement about the separating property and cyclicity follows from this decomposition and Lemma 3.1. $\square$

Next let us consider translations and dilations on each fibre on the null plane. Let $C:{{\mathbb {R}}}^{D-1}\rightarrow {{\mathbb {R}}}$ be a continuous function. We define the distorted lightlike translations as the following unitary operator on $L^2({{\mathbb {R}}}^D ,d\theta 'd\pmb {x}_\perp )$ (and with the identification of ${\mathfrak {H}}_m$ with it through the spacial decomposition $V_{\mathrm S}$):

15 $$\begin{aligned} (T_C \xi )(\theta ',\pmb {x}_\perp ) = e^{iC(\pmb {x}_\perp )e^{-\theta '}} \xi (\theta ', \pmb {x}_\perp ). \end{aligned}$$

Similarly, we consider the distorted lightlike dilations

16 $$\begin{aligned} (D_C\xi )(\theta ', \pmb {x}_\perp ) = \xi (\theta ' - C(\pmb {x}_\perp ), \pmb {x}_\perp ). \end{aligned}$$

If $C = \alpha$ is constant, they coincide with the usual translation and the dilation, respectively, see (12).

#### Remark 3.9

Note that since *C* is a continuous function *C*, $x_\perp \mapsto e^{iC(\pmb {x}_\perp )te^{-\theta '}}$ is a measurable vector field of bounded operators, that is, $\int _{{{\mathbb {R}}}^{D-1}}^\oplus e^{iC(\pmb {x}_\perp )te^{-\theta '}}\xi (\pmb {x}_\perp )d\pmb x_\perp \in {\mathfrak {H}}_m$ for all $\xi \in {\mathfrak {H}}_m$, with the identification (10). As an intermediate step, if *C* is a characteristic function, the claim is obvious. For an arbitrary continuous *C*, there is a family of simple functions $\{C_N\}_{N\in {{\mathbb {N}}}}$ converging uniformly to pointwise to *C*. Then $e^{iC_N(\pmb {x}_\perp )te^{-\theta '}}$ converges to $e^{iC(\pmb {x}_\perp )te^{-\theta '}}$ in the strong operator topology. $D_C$ is also a decomposable operator since $(D_C\xi )(\theta ',\pmb {x}_\perp )=\int _{{{\mathbb {R}}}^{D-1}}^\oplus D_C(\pmb {x}_\perp ) \xi (\theta ',\pmb {x}_\perp )d\pmb {x}_\perp$, where $D_C(\pmb {x}_\perp ) \xi (\theta ',\pmb {x}_\perp )= \xi (\theta ' - C(\pmb {x}_\perp ), \pmb {x}_\perp )$, and one can analogously prove that $x_\perp \mapsto D_C(\pmb {x}_\perp )$ is a measurable vector field of bounded operators passing to the $\theta '$-Fourier transform. In particular, since $H=\int _{{{\mathbb {R}}}^{D-1}}^{\oplus _{{\mathbb {R}}}} H(\pmb {x}_\perp )d\pmb {x}_\perp \subset {\mathfrak {H}}_m$ is a standard subspace, the subspaces $T_C H=\int _{{{\mathbb {R}}}^{D-1}}^{\oplus _{{\mathbb {R}}}} T_C(\pmb {x}_\perp )H(\pmb {x}_\perp )d\pmb {x}_\perp$ and $D_CH=\int _{{{\mathbb {R}}}^{D-1}}^\oplus D_C(\pmb {x}_\perp )H(\pmb {x}_\perp )d\pmb {x}_\perp$ are well-defined and standard as well.

#### Proposition 3.10

The family of real subspaces *H*(*R*), indexed by open connected regions $R\subset X_-^0$, is covariant with respect to $T_C, D_C$ for a continuous function *C*, i.e.

$$\begin{aligned} T_C H(R) = H(R+C)\quad \text {and} \quad D_{C} H(R) = H(e^{C}\cdot R), \end{aligned}$$

where $$R + C = \{(x_+ + C(\pmb {x}_\perp ), \pmb {x}_\perp ): (x_+, \pmb {x}_\perp ) \in R\}$$ and $$C\cdot R = \{(e^{C(\pmb {x}_\perp )}x_+, \pmb {x}_\perp ): (x_+, \pmb {x}_\perp ) \in R\}$$.

#### Proof

Let $g_0$ be a thin test function as in (13). The fibre-wise deformed test function $g_{{\mathfrak {t}}_C}(x_+, \pmb {x}_\perp ) = g(x_+ - C(\pmb {x}_\perp ), \pmb {x}_\perp )$ corresponds to the following one-particle vector:

$$\begin{aligned} {{\hat{g}}}_{{\mathfrak {t}}_C}(p_-, \pmb {p}_\perp ) = e^{iC(\pmb {x}_\perp )p_-}({{\hat{g}}})(p_-, \pmb {p}_\perp ), \end{aligned}$$

Therefore, we obtain the equality $V_{\mathrm S}(E g_{0,{\mathfrak {t}}_C})=T_CV_{\mathrm S}(Eg_0)$ once we prove that $H(R+C)$ contains the vectors which are the Fourier transform of $g_{0,{\mathfrak {t}}_C}(x_+, x_-, \pmb {x}_\perp ) = \delta (x_-) g_{{\mathfrak {t}}_C}(x_+, \pmb {x}_\perp )$ where $g_{{\mathfrak {t}}_C}$ is of the form $g(x_+ - C(\pmb {x}_\perp ), \pmb {x}_\perp )$ with a smooth *g*.

Indeed, we can mollify $g_{{\mathfrak {t}}_C}$ by an approximate delta function $\delta _n$ on $X_-^0$: $\delta _n$ is a positive smooth function with $\int \delta _1(x_+,\pmb {x}_\perp ) dx_+ d\pmb {x}_\perp = 1$ over the Lebesgue measure and $\delta _n(x_+,\pmb {x}_\perp ) = n^D \delta _1(nx_+, n\pmb {x}_\perp )$. The mollified function $g_{{\mathfrak {t}}_C} * \delta _n$ is smooth, supported in $R+C$ for sufficiently large *n* and its $x_+$ integral vanishes. Therefore, its Fourier transform belongs to $H(R+C)$ when restricted to the mass shell, and is equal to the Fourier transform of $g_{{\mathfrak {t}}_C}$ multiplied by that of $\delta _n$. The Fourier transform of $\delta _n$ is bounded and converge to 1, therefore, it converges to $Eg_{0,{\mathfrak {t}}_C}$ where $g_{0,{\mathfrak {t}}_C}(x_+, x_-, \pmb {x}_\perp ) = \delta (x_-)g_{0,{\mathfrak {t}}_C}(x_+, \pmb {x}_\perp )$. As $H(R+C)$ is closed, it contains $Eg_{0,{\mathfrak {t}}_C}$.

Similarly, for $g_{{\mathfrak {d}}_{C}}(x_+, \pmb {x}_\perp ) = g(e^{-C(\pmb {x}_\perp )}x_+ , \pmb {x}_\perp )$, its Fourier transform on $x_+$ for fixed $\pmb {x}_\perp$ as a function of $p_-$ is dilated by $e^{C(\pmb {x}_\perp )}$ and hence

$$\begin{aligned} (V_{\mathrm S}{{\hat{g}}}_{{\mathfrak {d}}_{C}})(\theta ', \pmb {x}_\perp ) = (V_{\mathrm S}{{\hat{g}}})(\theta '-C(\pmb {x}_\perp ), \pmb {x}_\perp ), \end{aligned}$$

that is, it holds that $V_{\mathrm S}(Eg_{0,{\mathfrak {d}}_{C}}) = D_{C} V_{\mathrm S}(Eg_0)$. Similarly as in the case of $g_{{\mathfrak {t}}_C}$, $Eg_{0,{\mathfrak {d}}_C} \in H(e^{C}\cdot R)$, where $g_{0,{\mathfrak {d}}_C}(x_+, x_-, \pmb {x}_\perp ) = \delta (x_-)g_{{\mathfrak {d}}_{C}}(x_+, \pmb {x}_\perp )$, we obtain the desired covariance. $\square$

Proposition 3.10 allows to generalize the disintegration (14) to more general open regions.

#### Proposition 3.11

Let *R* be a region on $X_-^0$ of the form $R = \{(x_+, \pmb {x}_\perp ): \pmb {x}_\perp \in I_\perp , C_1(\pmb {x}_\perp )< x_+ < C_2(\pmb {x}_\perp )\}$ for an open region $I_\perp \subset {{\mathbb {R}}}^{D-1}$ and two continuous functions $C_1, C_2:{{\mathbb {R}}}^{D-1}\rightarrow {{\mathbb {R}}}$. Then through the spatial decomposition we have

$$\begin{aligned} H(R) \simeq \int ^{\oplus _{{\mathbb {R}}}}_{I_\perp } H_{U(1)}\left( (C_1(\pmb {x}_\perp ),C_2(\pmb {x}_\perp ))\right) d^{D-1}\pmb {x}_\perp . \end{aligned}$$

Similarly, the subspace of a null-cut decomposes:

$$\begin{aligned} H(N_C)&\simeq \int ^{\oplus _{{\mathbb {R}}}}_{{{\mathbb {R}}}^{D-1}}H_{U(1)}((C(\pmb {x}_\perp ), \infty ))d^{D-1}\pmb {x}_\perp , \\ H(N_{C}^\dagger )&\simeq \int ^{\oplus _{{\mathbb {R}}}}_{{{\mathbb {R}}}^{D-1}} H_{U(1)}\left( (-\infty , C(\pmb {x}_\perp )) \right) d^{D-1}\pmb {x}_\perp , \end{aligned}$$

where $N_{C}^\dagger$ is the interior of the complement of the null-cut $N_{C}$ on the null-plane:

$$\begin{aligned} N_C^\dagger&=X_-^0\setminus \overline{N_C} =\{ x_+<C(\pmb {x}_\perp ),x_-=0, \pmb {x}_\perp \in {\mathbb {R}}^{D-1} \}^\circ . \end{aligned}$$

#### Proof

Recall that by Proposition 3.8 the decomposition holds for the case $R = (0,1)\times I_\perp$. We can dilate this by $D_{C_2-C_1}$ and then translate it by $C_1$ to arrive at *R* in the desired form. By the covariance of the whole family $\{H(R)\}$ with respect to fibre-wise translations and dilations by Proposition 3.10, we obtain the desired decomposition.

If we start from the rectangle ${{\mathbb {R}}}_{+}\times {{\mathbb {R}}}^{D-1}$ (analogously for ${{\mathbb {R}}}_{-}\times {{\mathbb {R}}}^{D-1}$), then we deform the rectangle via the distorted lightlike translation $T_C$ to the null-cut $N_C$ or the interior of its complement $N_C^\dagger$, respectively. From the covariance of $T_C$ and Proposition 3.8, we obtain the decompositions:

17 $$\begin{aligned} H(N_{C})&\simeq \int ^{\oplus _{{\mathbb {R}}}}_{{{\mathbb {R}}}^{D-1}}H_{U(1)}((C(\pmb {x}_\perp ), \infty ))d^{D-1}\pmb {x}_\perp \nonumber \\ H(N_{C}^\dagger )&\simeq \int ^{\oplus _{{\mathbb {R}}}}_{{{\mathbb {R}}}^{D-1}} H_{U(1)}\left( (-\infty , C(\pmb {x}_\perp )) \right) d^{D-1}\pmb {x}_\perp . \end{aligned}$$

$$\square $$

### Duality for null cuts

#### Theorem 3.12

The standard subspaces $H(N_C)$ of continuous *C* satisfy duality (see Fig. 1):

$$\begin{aligned} H(N_C)=H(N_C)''=H(N_C''). \end{aligned}$$

#### Proof

The first equality follows from the fact that $H(N_C)$ is a standard subspace.

Next we show $H(N_C) \subset H(N_C'')$. Let *a* be a vector pointing in the positive $x_1$-direction (or the negative $x_-$ direction) we have $N_C \subset N_C'' - ta$ for $t>0$. Indeed, if $x \in N_C, y\in N_C'$, it has $x_- = 0$, and $(x - y)^2 = -2(x_+ - y_+)y_- - (\pmb {x}_\perp - \pmb {y}_\perp )^2 < 0$. Furthermore, as $x_+$ can be arbitrarily large, it must hold that $x_+ - y_+>0, y_->0$ and hence adding $-ta$ to *y* just decreases the first term. Therefore, for any $y \in N_C'$, $y-ta$ is spacelike from $x \in N_C$, that is, $N_C \subset N_C'' - ta$. Therefore, by Lemma 3.7, we have $H(N_C)\subset H(N_C'')$.

Lastly we prove $H(N_C'') \subset H(N_C)''$. It is easy to see that $N_C^\dagger - ta \subset N_C'$ for any $t>0$, where *a* is again a vector pointing in the positive $x_1$-direction, hence we have $H(N_C^\dagger ) \subset H(N_C')$ by Lemma 3.7. As we have $H(N_C'') \subset H(N_C')'$ by locality and we have $H(N_C'') \subset H(N_C')' \subset H(N_C^\dagger )' = H(N_C)''$, where the last equality follows from Lemma 3.1 and (17). $\square$

As an example, it is immediate to see that the causal completion of the zero null-cut $N_0 = N_{C_0}$ with $C_0(\pmb {x}_\perp ) = 0$ is the right wedge: $N_0''=W_1$, hence $H(N_0)=H(N_0'')=H(W_1)$.

While it is possible to define $H(N_C)$ for non continuous curves (see Sect. 6), the continuity condition Theorem 3.12 cannot be removed. Indeed, if $C=\chi _{{\mathbb {Q}}^{D-1}}$ is the characteristic function of ${\mathbb {Q}}^{D-1}$ then $H(N_C)=H(W_R)$ since the direct integrals

$$\begin{aligned} H(N_C)&=\int _{{{\mathbb {R}}}^{D-1}}^{\oplus _{{\mathbb {R}}}} H_\mathrm{U(1)}((\chi _{{\mathbb {Q}}^{D-1}}(\pmb {x}_\perp ),+\infty ))d^{D-1} \pmb {x}_\perp \\&=\int _{{{\mathbb {R}}}^{D-1}}^{\oplus _{{\mathbb {R}}}} H_\mathrm{U(1)}((0,+\infty ))d^{D-1}\pmb {x}_\perp =H(W_1) \end{aligned}$$

are equal up to a null measure set, but $H(N_C'')$ is strictly contained in $H(W_R)$ when the definition (5) is considered .

Let us define the deformed wedge for a continuous function $C:{{\mathbb {R}}}^{D-1}\rightarrow {{\mathbb {R}}}$ by

$$\begin{aligned} W_C:=\{\pmb {x}\in {{\mathbb {R}}}^{D+1},x_-<0,x_+>C(\pmb {x}_\perp )\}. \end{aligned}$$

Then we have:

#### Proposition 3.13

$$H(N_C)=H(W_C)$$.

#### Proof

It is clear from the definition that $N_C\subset W_C + tx_-$ for every $t>0$. From Lemma 3.7, it follows $H(N_C)\subset H(W_C)$.

For the opposite direction, we will deduce $H(W_C) \subset H(N_C'')$. The claim follows from isotony and the previous theorem. Let $y\in W_C$, then we can write $y = x + a$ with $x \in N_C$ and $a=(0,a_-,0)$ with $a_-<0$. It is enough to prove $N_C+t a \subset N_C''$ for $t > 0$, or equivalently, $N_C\subset N_C''-t a$ for every $t>0$. This is analogous to the proof of Theorem 3.12. $\square$

## Modular Operator for Null Cuts

The following is of an independent interest, because it is an assumption of the limited version of QNEC [CF20].

### Proposition 4.1

Let $C_1, C_2:{{\mathbb {R}}}^{D-1}\rightarrow {{\mathbb {R}}}$ be continuous functions such that $C_1(\pmb {x}_\perp ) < C_2(\pmb {x}_\perp )$. Then the inclusion $H(N_{C_2})\subset H(N_{C_1})$ is a HSMI.

### Proof

Note that, if $K \subset H$ is a HSMI and *U* is a unitary, then also $UK \subset UH$ is a HSMI by Lemma 2.1.

Recall that^1^$H(W_+) \subset H(W_1)$ is a HSMI, where $C_+(\pmb {x}_\perp ) = 1$ and $W_+ = W_{C_+}$ is the shifted wedge. Put also $C_0(\pmb {x}_\perp ) = 0$, so that $W_1 = W_{C_0}$. By fibre-wise covariance (Proposition 3.10), the inclusion $H(N_{C_2})\subset H(N_{C_1})$ is unitarily equivalent to $H(W_+) = H(C_+)\subset H(C_0) = H(W_1)$. Indeed, we can take $C_{\mathrm {dl}}(\pmb {x}_\perp ) = \log (C_2(\pmb {x}_\perp ) - C_1(\pmb {x}_\perp ))$ and $C_{\mathrm {tr}}(\pmb {x}_\perp ) = C_1(\pmb {x}_\perp )$. Then we have $T_{C_{\mathrm {tr}}}D_{C_{\mathrm {dl}}}H(W_1) = H(C_{\mathrm {tr}} + e^{C_{\mathrm {dl}}}C_0) = H(N_{C_1})$ and $T_{C_{\mathrm {tr}}}D_{C_{\mathrm {dl}}}H(W_+) = H(C_{\mathrm {tr}} + e^{C_{\mathrm {dl}}}C_+) = H(N_{C_2})$. Then the desired HSMI follows from this unitary equivalence. $\square$

In the rest of this Section, we will study the modular operator of the standard spaces $H(N_C)$. We call $Q_S$ the unitary that results from the concatenation of $V_{\mathrm S}$ (cf. Sect. 3.2) and the fibre-wise unitary between $L^2({{\mathbb {R}}},d\theta ')$ and the *U*(1)-current mentioned in Sect. 2.4.

### Proposition 4.2

The unitary operator $Q_S$ between ${\mathfrak {H}}_m$ and the direct integral of *U*(1)-current Hilbert spaces decomposes the modular operator of $C(N_0)$:

$$\begin{aligned} \log (\Delta _{H(N_0)})&=\log (\Delta _{H(W_R)})\\&={{\mathrm{Ad\,}}}_{Q_S}\int ^{\oplus }_{{{\mathbb {R}}}^{D-1}} \log (\Delta _{H_{U(1)}({{\mathbb {R}}}_+)})d^{D-1}\pmb {x}_\perp . \end{aligned}$$

### Proof

The causal complement of $N_0$ is the left wedge $W_L$:

$$\begin{aligned} N_0'&=\{x\in {{\mathbb {R}}}^{D+1}; x_+< 0,x_->0,\pmb {x}_\perp \in {{\mathbb {R}}}^{D-1}\}=W_L. \end{aligned}$$

By Theorem 3.12, we know $H(N_0)=H(N_0'')=H(W_R)$. By the Bisognano–Wichmann property (HK6) in Sect. 2.2, we know that the modular group of the right wedge coincides with the representation of the boost group along $x_1$:

$$\begin{aligned} \Delta _{H(W_R)}^{it}= U_m(\Lambda _{1}(-2\pi t)). \end{aligned}$$

By (16) the representation of the boost group is equivalent to the constant dilation group of the *U*(1)-current on each fibre of the spatial decomposition of ${\mathfrak {H}}_m$ and hence:

$$\begin{aligned} {{\mathrm{Ad\,}}}_{Q_S}\Delta _{H(W_R)}^{-it}=\int ^{\oplus }_{{{\mathbb {R}}}^{D-1}} U_\mathrm{U(1)}(2\pi t,0)\,d^{D-1}\pmb {x}_\perp . \end{aligned}$$

The statement follows from Proposition A.1 and the Bisognano–Wichmann property for the *U*(1)-current (cf. Sect. 2.4). $\square$

The standard subspace of the constant null cut $C_s(\pmb {x}_\perp )=s$ is

$$\begin{aligned} H(N_{s})&=T_{C_s}H(N_0) =U_m(1, {\mathfrak {t}}_{x_+}(s))H(W_1) =H({\mathfrak {t}}_{x_+}(s)W_1). \end{aligned}$$

That means that $H(N_s)$ equals the standard subspace associated with the wedge ${\mathfrak {t}}_{x_+}(s)W_1$. According to the Bisognano–Wichmann property, the associated modular group is the boost group leaving the wedge ${\mathfrak {t}}_{x_+}(s)W_1$ invariant:

$$\begin{aligned} \Delta _{H(N_s)}^{it}&= U_m(1,{\mathfrak {t}}_{x_+}(s))\Delta _{H(N_0)}^{it}U_m(1,{\mathfrak {t}}_{x_+}(s))^*\\&=U_m(1,{\mathfrak {t}}_{x_+}((1-e^{2\pi t})s))\Delta _{H(N_0)}^{it}. \end{aligned}$$

From this, we have the following well-known (cf. Lemma 2.1) relation between generators.

18 $$\begin{aligned} \log {\Delta _{H(N_s)}}=\log {\Delta _{H(N_0)}}+2\pi sP, \end{aligned}$$

where *P* is the generator of translations $U_m({\mathfrak {t}}_{x_+}(s))$ along the light ray $x_+$.

The formula (18) has a natural generalization to arbitrary null-cuts. The key structures that we will utilize are half-sided modular inclusion of certain null-cut subspaces. In the following, we will use the notation for half-sided modular inclusions introduced in Sect. 2.1.

**Fig. 2:**
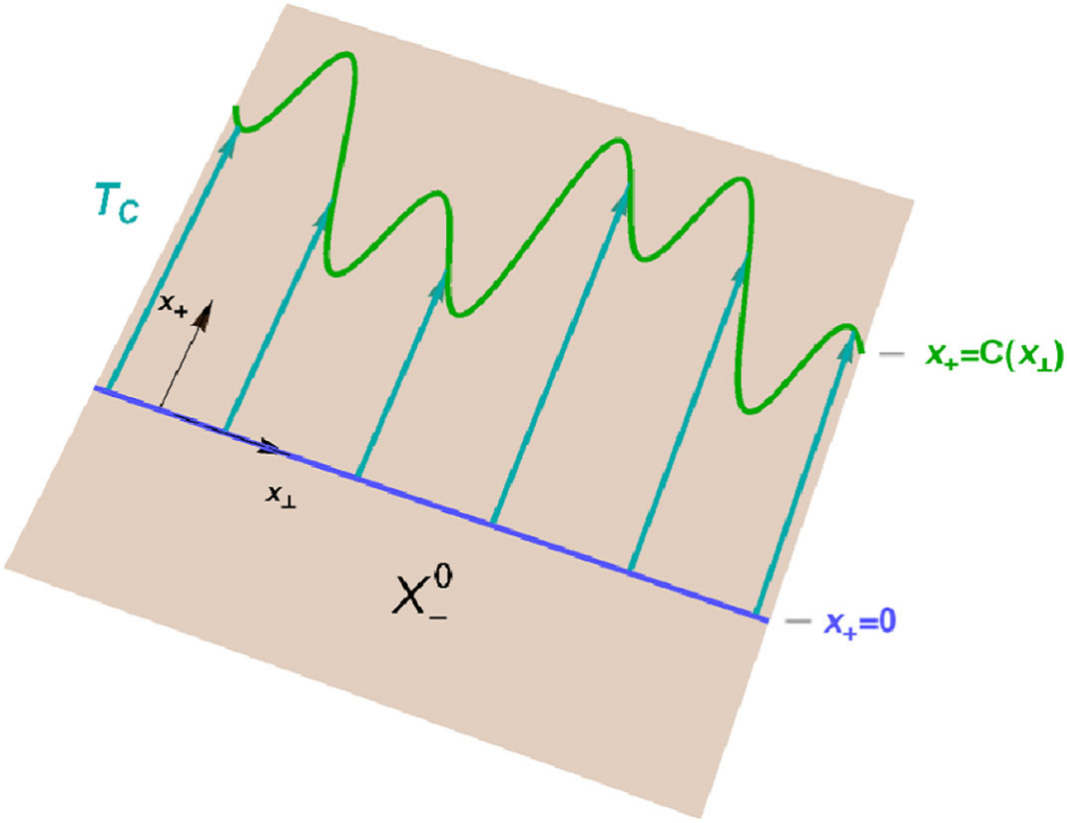
Geometric action of distorted lightlike translations

Now we state one of our main results (the idea of the proof is visualised in Fig. 2).

### Theorem 4.3

Let $C:{{\mathbb {R}}}^{D-1}\rightarrow {{\mathbb {R}}}$ be a continuous function, then the generator of the modular group of the associated null-cut is decomposed as follows, where the equivalence is given by the operator $Q_{\mathrm S}$ of Proposition 4.2.

19 $$\begin{aligned} \log (\Delta _{H(N_C)})\simeq \int ^\oplus _{{{\mathbb {R}}}^{D-1}}\left( \log (\Delta _{H_{U(1)}({{\mathbb {R}}}_+)})+ 2\pi C(\pmb {x}_\perp ) P_{\pmb {x}_\perp }\right) \,d\pmb {x}_\perp , \end{aligned}$$

where $P_{\pmb {x}_\perp }$ is the generator of translations on each fibre.

### Proof

As we have $N_C = T_C N_0$ (with the notations of Lemma 3.7), we have $\Delta _{H(N_C)}^{it} = {{\mathrm{Ad\,}}}T_C (\Delta _{H(N_0)}^{it})$. Note that $T_C$ is a decomposable operator implementing the fiber-wise translations, therefore by the decomposition through $Q_\mathrm {S}$,

$$\begin{aligned} \Delta _{H(N_C)}^{it}&\simeq \int ^\oplus _{{{\mathbb {R}}}^{D-1}} {{\mathrm{Ad\,}}}U_{\mathrm{U(1)}}(0,{\mathfrak {t}}(C(\pmb {x}_\perp ))(\Delta _{H_{U(1)}({{\mathbb {R}}}_+)}^{it}) \,d\pmb {x}_\perp \\&=\int ^\oplus _{{{\mathbb {R}}}^{D-1}}\exp \left( it\left( \log (\Delta _{H_{U(1)}({{\mathbb {R}}}_+)})+ 2\pi C(\pmb {x}_\perp ) P_{\pmb {x}_\perp }\right) \right) \,d\pmb {x}_\perp , \end{aligned}$$

where the second equation is the fibre-wise transformation law of a simple HSMI (Theorem 2.3). By Proposition A.1 and the Stone theorem, this relation passes to the generators.

$$\square $$

Let us comment on the second quantized net $({{\mathcal {A}}}, \mathrm {\Gamma }_+(U_m), {\mathcal {F}}_+({\mathfrak {H}}_m))$. By Proposition 2.4, a HSMI of standard subspaces promotes to a HSMI of von Neumann algebras, hence the following is an immediate consequence of Proposition 4.1 and Theorem 3.12.

### Corollary 4.4

Let $C_1, C_2:{{\mathbb {R}}}^{D-1}\rightarrow {{\mathbb {R}}}$ be continuous functions such that $C_1(\pmb {x}_\perp ) < C_2(\pmb {x}_\perp )$. Then the inclusion ${{\mathcal {A}}}(N_{C_2}'')\subset {{\mathcal {A}}}(N_{C_1}'')$ is a HSMI.

It is not easy to write the second-quantized version of (19): while the second quantization of the left-hand side is straightforward $\mathrm {d\Gamma }_+(\log \Delta _{H(N_C)}) = \log \Delta _{{{\mathcal {A}}}(N_C''),\Omega }$, where $\mathrm {d\Gamma }_+(A) = 0\oplus A \oplus (A\otimes {\mathbbm {1}} \oplus {\mathbbm {1}}\otimes A)\cdots$ is the additive second quantization, the direct-integral structure on the right-hand side translates to the *continuous tensor product* structure in the second quantization [AW66, Nap71]. Conceptually, it should be an “integral” of the second-quantized generators on fibres, but we do not know how to formulate such an integral corresponding to (1). Instead, we were able to formulate the HSMI without reference to the (undefined) stress-energy tensor smeared on the null plane.

On the other hand, for general interacting models, we do not have this simple second-quantization structure, and the validity of HSMI for the inclusions of null cut regions remains open, see Sect. 6.

## Relative Entropy and Energy Bounds

### Decomposition of the relative entropy for coherent states

We recall that the formula for Araki relative entropy of a von Neumann algebra ${{\mathcal {A}}}\subset {\mathcal {B}}({{\mathcal {K}}})$ with respect to two vector states $\omega _1$ and $\omega _2$ is given by

$$\begin{aligned} S(\omega _1||\omega _2)=-\langle \xi ,\log \Delta _{\eta ,\xi } \xi \rangle \end{aligned}$$

where $\xi ,\eta \in {{\mathcal {K}}}$ implement $\omega _1$ and $\omega _2$ on ${{\mathcal {A}}}$, respectively and $S_{\eta ,\xi } = J_{\eta ,\xi }\Delta _{\eta ,\xi }^\frac{1}{2}$ is the relative Tomita opereator:

$$\begin{aligned} S_{\eta ,\xi }:{{\mathcal {A}}}\xi \ni a\xi \mapsto a^*\eta \in {{\mathcal {A}}}\eta . \end{aligned}$$

It is immediate to see that if $\eta =\xi$, then $\Delta _{\xi ,\xi }$ is the modular operator of ${{\mathcal {A}}}$ with respect to $\xi$ and $S(\omega _1||\omega _1)=0$.

Let $H \subset {{\mathcal {H}}}$ be a standard subspace and $\mathrm {w(\psi )}$ be the Weyl operator of $\psi \in {{\mathcal {H}}}$ on $\Gamma _+({{\mathcal {H}}})$. A state on a von Neumann algebra ${{\mathcal {A}}}\subset {{\mathcal {B}}}(\Gamma _+({{\mathcal {H}}}))$ is called **coherent** if it is given by $\omega _\psi (\cdot ):=\omega (\mathrm w(\psi )\cdot \mathrm w(\psi )^*)$, where $\omega$ is the Fock vacuum.

Following [CLR20], given a standard subspace $H\subset {{\mathcal {H}}}$, we can consider the following quantity

$$\begin{aligned} S_H(\psi )=\mathrm {Im}\,\langle \psi ,P_Hi\log \Delta _H \psi \rangle , \end{aligned}$$

where $P_H$ is an unbounded real projection called *cutting projection*. The cutting projection is determined by the modular operator and modular conjugation of *H*. Its explicit form is:

$$\begin{aligned} P_H=a(\Delta _H)+ J_Hb(\Delta _H) \end{aligned}$$

where $$a(\lambda )=\lambda ^{-1/2}(\lambda ^{-1/2}-\lambda ^{1/2})^{-1}$$ and $$b=(\lambda ^{-1/2}-\lambda ^{1/2})^{-1}$$.

If $$h\in H$$, then

$$\begin{aligned} S_H(\psi )=-\langle \psi ,\log \Delta _H \psi \rangle . \end{aligned}$$

We have the following relation with the relative entropy of second quantization algebra:

$$\begin{aligned} S_{R(H)}(\omega _\psi ||\omega )=S_H(\psi ). \end{aligned}$$

where $S_{R(H)}(\omega _\psi ||\omega )$ is the relative entropy of $\omega _\psi$ and $\omega$ with respect to *R*(*H*) [CLR20,  Proposition 4.2].

#### Lemma 5.1

Consider a standard subspace *H* that is a direct integral of standard subspaces $H=\int _X^{\oplus _{{\mathbb {R}}}} H(x)d\nu (\lambda )\subset \int _X^\oplus {{\mathcal {K}}}_\lambda d\nu (\lambda )$ with decomposable modular generator $\log (\Delta _H)=\int ^\oplus _X \log (\Delta _{H(\lambda )})d\nu (\lambda )$. Moreover, let $\psi =\int ^\oplus _X \psi (\lambda )d\nu (\lambda )\in {{\mathcal {K}}}$. Then the relative entropy of $\omega _\psi$ and the vacuum with respect to *H* decomposes:

$$\begin{aligned} S_{R(H)}(\omega _\psi ||\omega )&=\int _X S_{R(H(\lambda ))}(\omega _{_\Psi (\lambda )}(\lambda )||\omega (\lambda ))d\nu (\lambda ), \end{aligned}$$

where $\omega (\lambda )$ denotes the vacuum on the Fock space of ${{\mathcal {K}}}_\lambda$.

#### Proof

Following the previous arguments, we have $S_{R(H)}(\omega _\psi ||\omega )=S_H(\psi )$. By assumption the modular generator decomposes. It is straightforward to check that $J_H=\int ^\oplus _X J_{H(\lambda )}d\nu (\lambda )$ decomposes as well. Applying Proposition A.1, the cutting projection decomposes accordingly $P_H=\int ^\oplus _X P_{H(\lambda )}\mu (\lambda )$. Thus

$$\begin{aligned} \langle \psi ,P_Hi\log \Delta _H \psi \rangle =\int _X \langle \psi (\lambda ),P_{H(\lambda )}i\log (\Delta _{H(\lambda )})\psi (\lambda ) \rangle d\nu (\lambda ), \end{aligned}$$

in particular

$$\begin{aligned} S_H(\psi )&=\mathrm {Im}\,\int _X \langle \psi (\lambda ),P_{H(\lambda )}i\log (\Delta _{H(\lambda )})\psi (\lambda ) \rangle d\nu (\lambda )\\&=\int _X\mathrm {Im}\,\langle \psi (\lambda ),P_{H(\lambda )}i\log (\Delta _{H(\lambda )})\psi (\lambda ) \rangle d\nu (\lambda )=\int _X S_{H(\lambda )}(\psi (\lambda )). \end{aligned}$$

Since $S_{H(\lambda )}(\psi (\lambda ))=S_{R(H(\lambda ))}(\omega _{\psi (\lambda )}(\lambda )||\omega (\lambda ))$, we conclude the argument. $\square$

#### Remark 5.2

The modular operator of the direct integral of standard subspaces always decomposes into the direct integral of modular operators of the fibre subspaces. This can be seen from the KMS condition for standard subspaces (see [Lon08,  Proposition 2.1.8]) and the results in “Appendix A”.

### Relative entropy for coherent states on the null plane

For a single *U*(1)-current net, an explicit formula for the relative entropy was computed in [Lon20], where the *U*(1)-current (cf. Sect. 2.4) is differently, but equivalently, defined. To be precise, a test function in [Lon20] is replaced by its primitive in Sect. 2.4. We translate the results of [Lon20] to the definition of the *U*(1)-current introduced in Sect. 2.4.

Let $I_t=(t,+\infty )\subset {{\mathbb {R}}}$, $H_\mathrm{U(1)}(I_t)$ be its standard subspace and ${{\mathcal {A}}}(I_t)=R(H_\mathrm{U(1)}(I_t))$ be the second quantization von Neumann algebra. Consider the map $\beta _k(\mathrm w(\xi ))=e^{-i\int _{{\mathbb {R}}}k(x)L(x)\,dx}\mathrm w(\xi )$ with $k,l\in C_0^\infty ({{\mathbb {R}}},{{\mathbb {R}}})$, ${\hat{l}}(0)=0$, $C_0^\infty ({{\mathbb {R}}},{{\mathbb {R}}})\ni L(x)=\int _{-\infty }^xl(s)ds$ the primitive of *l* and $\xi \in H_\mathrm{U(1)}(I_t)$ being the one-particle vector associated to *l*. Then $\beta _k$ extends to an automorphism of the local algebra ${{\mathcal {A}}}(I_t)$ - a so-called Buchholz-Mack-Todorov (BMT) automorphism [BMT88]. We denote this extension by $\beta _k$ as well. One has [Lon20,  Theorem 4.7]:

$$\begin{aligned} S_{{{\mathcal {A}}}(I_t)}(\omega \circ \beta _k||\omega )=\pi \int _t^{+\infty }(x-t) k^2(x)dx, \end{aligned}$$

where $\omega$ is the vacuum state on the *U*(1)-current.

We can generalize BMT automorphisms to the direct integral of *U*(1)-currents. Let $h,g\in C_0^\infty (X_-^0,{{\mathbb {R}}})$ with $\hat{g}(0, \pmb {x}_\perp ) = 0$ where $\hat{g}$ is the fiberwise Fourier transform, *G* the fiberwise primitive of *g* and $\xi =V_SE\delta (x_-)g$, we define the automorphism

$$\begin{aligned} \beta _h(\mathrm w(\xi ))=e^{-i\int _{X_-^0} h(x)G(x)\,dx}\mathrm w(\xi ). \end{aligned}$$

Let us consider the relative entropy between $\omega$ and $\omega \circ \beta _h$ with respect to local algebras of null cuts:

#### Proposition 5.3

We have

$$\begin{aligned} S_{R(H(N_C))}(\omega \circ \beta _h||\omega )=\pi \int _{{{\mathbb {R}}}^{D-1}}\int _{C(\pmb {x}_\perp )}^\infty (x_+-C(\pmb {x}_\perp )) h(x_+,\pmb {x}_\perp )^2 dx_+ d\pmb {x}_\perp . \end{aligned}$$

#### Proof

As the support of *h* is compact, *C* is bounded on the restriction to $\pmb {x}_\perp$ of the support of *h*, and by adding an appropriate smooth function supported in $N_C^\dagger$, we may assume that $\int h(x_+,\pmb {x}_\perp )dx_+=0$ for every $\pmb {x}_\perp$ without changing the state $\omega \circ \beta _h$ on $R(H(N_C))$. Then $\delta (x_-)h$ is a thin test function and we call ${\mathfrak {h}}:=V_SE\delta (x_-)h$ its one-particle vector in the spatial decomposition.

In this case, the BMT automorphism $\beta _h$ is generated by the adjoint action of the Weyl operator $\mathrm w({\mathfrak {h}})$. The symplectic form can be computed fibrewise using (7)

20 $$\begin{aligned}&{{\mathrm{Ad\,}}}\mathrm w({\mathfrak {h}}) \left( \mathrm w(\xi )\right) =e^{2i \mathrm {Im}\,\langle {\mathfrak {h}},\xi \rangle }\mathrm w(\xi ). \end{aligned}$$

21 $$\begin{aligned}&\mathrm {Im}\,\langle {\mathfrak {h}},\xi \rangle = \int _{{{\mathbb {R}}}^{D-1}} \mathrm {Im}\,\langle {\mathfrak {h}}(\pmb {x}_\perp ),\xi (\pmb {x}_\perp ) \rangle _{U(1)}d\pmb {x}_\perp =\frac{1}{2}\int _{{{\mathbb {R}}}^{D}} h(x_+,\pmb {x}_\perp )G(x_+,\pmb {x}_\perp )dx_+d\pmb {x}_\perp . \end{aligned}$$

Thus, on $R(H(N_C))$, $\omega \circ \beta _h$ is the coherent state $\omega _{{\mathfrak {h}}}$ and we can apply Lemma 5.1:

$$\begin{aligned} S_{R(H(N_C))}(\omega _{{\mathfrak {h}}}||\omega )=\int _{{{\mathbb {R}}}^{D-1}} S_{{{\mathcal {A}}}(I_{C(\pmb {x}_\perp )})}(\omega _{{\mathfrak {h}}(\pmb {x}_\perp )}(\pmb {x}_\perp )|| \omega _{\pmb {x}_\perp })d\pmb {x}_\perp . \end{aligned}$$

The coherent state $\omega _{{\mathfrak {h}}(\pmb {x}_\perp )}(\pmb {x}_\perp )$ on the *U*(1) current is generated by the adjoint action of $\mathrm w_{U(1)}({\mathfrak {h}}(\pmb {x}_\perp ))$ on the fibrewise vacuum $\omega (\pmb {x}_\perp )$. This in turn coincides with the action of the BMT automorphism $\beta _{h(\pmb {x}_\perp )}$ on the *U*(1) vacuum $\omega ({\pmb {x}_\perp })$. Therefore, we can insert the results for a single *U*(1)-current and have:

$$\begin{aligned} S_{R(H(N_C))}(\omega \circ \beta _h||\omega )&=\int _{{{\mathbb {R}}}^{D-1}}S_{{{\mathcal {A}}}(I_{C(\pmb {x}_\perp )})}(\omega ({\pmb {x}_\perp }) \circ \beta _{h(\pmb {x}_\perp )}||\omega ({\pmb {x}_\perp }))d\pmb {x}_\perp \\&=\pi \int _{{{\mathbb {R}}}^{D-1}}\int _{C(\pmb {x}_\perp )}^\infty (x_+-C(\pmb {x}_\perp )) h(x_+,\pmb {x}_\perp )^2 dx_+ d\pmb {x}_\perp . \end{aligned}$$

$$\square $$

### The ANEC and the QNEC

One of the motivations to study quantum energy conditions comes from energy constraints in General Relativity, see e.g. [Few12]. For example, the pointlike positivity of the stress energy tensor along null-directions is called Null Energy Condition (NEC). It is often used in General Relativity to model matter distributions and it plays an important role in the discussion of singularity theorems. In QFT, however, it is not difficult to see that the stress energy tensor smeared by a positive test function can not be positive but only bounded from below, see e.g. [FH05].

One candidate for an analogous expression of the NEC in QFT is the Averaged Null Energy Condition (ANEC). It claims that the the integral of the expectation value of the energy-momentum tensor in any physical state, along any complete, lightlike geodesic $\gamma$ is always non-negative (see e.g. [FLPW16,  (3)], [Ver00]):

22 $$\begin{aligned} \langle \Phi |\int _{{\mathbb {R}}}ds \,T_{\mu ,\nu }(\gamma (s))k^\mu k^\nu \Phi \rangle \ge 0, \end{aligned}$$

where $k^\mu$ is the tangent of $\gamma .$ We can write $T_{++}$ when we consider the null $x_+$-direction $(x_+,0,\pmb {0})$. It is expected that (22) is satisfied for a dense set of vectors in a certain formal sense.

Another candidate is the Quantum Null Energy Condition (QNEC). It is a local bound on the expectation value of the null energy density given by the von Neumann entropy of the null cut (the following is only symbolic and we do not attempt to justify the expressions):

23 $$\begin{aligned} \langle T_{++}(x) \rangle _\rho \ge \frac{1}{2\pi }S''_{R(N_C)}(\rho ), \end{aligned}$$

where $S_{R(N_C)}(\rho )$ is the von Neumann entropy of a state $\rho$ with respect to the local algebra $R(N_C)$ of a null cut $N_C$, *x* is on the boundary of the cut *C*, and the derivative is taken in the sense of the deformation of *C* at the point *x* (see [BFKW19]). The local algebras in AQFT are usually of type III and the von Neumann entropy is infinite (see e.g. [OP04]). That is why (23) can only be considered as a formal expression.

Under the (formal) assumption that the local algebra $R(N_C)$ is of type I, in standard form and with the associated modular operator as in (1), [BFLW16,  Sec. 5.3] argued, for null cuts $N_C$ and in [KLLSM18] for deformed wedges $W_C$, that the QNEC can be reformulated as convexity of the relative entropy:

24 $$\begin{aligned} S_{R(N_C)}''(\rho ||\omega )\ge 0, \end{aligned}$$

where the second derivative is considered w.r.t. to positive deformations of the lower boundary of the null cut.

We will review the (not completely rigorous) arguments. The form of the modular Hamiltonian (1) plays an important role. To justify that we consider the QNEC for the free scalar field as convexity of the relative entropy (24) in the following, it is, therefore, important that the modular operator $\log (\Delta _{H(N_C)})$ in (19) is a clear analogue of the modular Hamiltonian in (1).

Let $\omega$ be the vacuum, $\rho$ a density matrix and $\left. \rho \right| _{N_C}$ the normal state it generates on $R(N_C)$. In the physics literature, the relative entropy of $\omega$ and $\rho$ w.r.t. $R(N_C)$ is expressed in terms of the (vacuum) modular Hamiltonian $\widetilde{H_C}$ on the future horizon of the null cut $N_C$ [KLLSM18,  (22)]:

$$\begin{aligned} S_{R(N_C)}(\rho ||\omega )=\langle \xi _\rho ,\widetilde{H_C} \xi _\rho \rangle -S(\left. \rho \right| _{N_C}). \end{aligned}$$

In the above formula, $\xi _\rho$ is a vector representative of $\left. \rho \right| _{N_C}$ and $\widetilde{H_C}$ has the form (cf. [CTT17b,  (1.5)]):

$$\begin{aligned} \widetilde{H_C} = 2\pi \int d^{D-1}\pmb {x}_\perp \int _{C(\pmb {x}_\perp )}^\infty d\lambda (\lambda - C(\pmb {x}_\perp ))T_{++}(\lambda ,\pmb {x}_\perp ). \end{aligned}$$

The key is the formal connection between $\widetilde{H_C}$ and the stress-energy tensor $T_{++}$. Consider deformations of the lower boundary $C(\pmb {x}_\perp )\rightarrow C(\pmb {x}_\perp )+t A(\pmb {x}_\perp )$ with the deformation parameter $t\in {{\mathbb {R}}}$. The second formal variational derivative of $\widetilde{H_{C+tA}}$ with respect to *t* is [KLLSM18,  (14)]:

$$\begin{aligned} \left. \frac{d^2}{dt^2}\right| _{t=0}\widetilde{H_{C+tA}}= \int _{{{\mathbb {R}}}^{D-1}}A(\pmb {x}_\perp )^2 T_{++}(C(\pmb {x}_\perp ),\pmb {x}_\perp )d^{D-1}\pmb {x}_\perp . \end{aligned}$$

With this connection, the following equivalence holds independently of the choice of the deformation function $A(\pmb {x}_\perp )$ [KLLSM18,  IV.B]:

$$\begin{aligned} \int _{{{\mathbb {R}}}^{D-1}}A(\pmb {x}_\perp )^2 \langle T_{++} \rangle _\rho d^{D-1}\pmb {x}_\perp \ge \left. \frac{d^2}{dt^2}\right| _{t=0}S(\left. \rho \right| _{N_{C+tA}})&\Leftrightarrow \frac{d^2}{dt^2} \left( \langle \widetilde{H_{C+tA}} \rangle _\rho -S(\left. \rho \right| _{N_{C+tA}})\right) \ge 0 \\&\Leftrightarrow \frac{d^2}{dt^2}S_{R(N_{C+tA})}(\rho ||\omega )\ge 0. \end{aligned}$$

The authors of [KLLSM18] define $S(\left. \rho \right| _{N_C})''({{\textbf {y}}}_\perp ):=\lim \limits _{A^2(\pmb {x}_\perp )\rightarrow \delta (\pmb {x}_\perp -{{\textbf {y}}}_\perp )} \left. \frac{d^2}{dt^2}\right| _{t=0}S(\left. \rho \right| _{N_{C+tA}})$ and $S_{R(N_C)}(\rho ||\omega )''({{\textbf {y}}}_\perp )$ analogously. In this sense, we have:

$$\begin{aligned} \langle T_{++} \rangle _\rho (C({{\textbf {y}}}_\perp ),{{\textbf {y}}}_\perp )\ge S(\left. \rho \right| _{N_C})''({{\textbf {y}}}_\perp ) \Leftrightarrow S_{R(N_C)}(\rho ||\omega )''({{\textbf {y}}}_\perp )\ge 0. \end{aligned}$$

Since this holds independently of ${{\textbf {y}}}_\perp$, it implies the formal equivalence of (23) and (24).

Now, the QNEC in its relative entropy form (24) can be investigated on a rigorous level because the relative entropy has a meaningful definition for von Neumann algebras of any type (see e.g. [OP04]). The QNEC (24) has already been verified for some families of AQFT models and states (see e.g. [Lon20, CLR20, Lon19, Pan20]) and is expected to hold in general in the AQFT context.

In [CF20], the authors assume that the inclusions of null cut algebras are HSMI and prove an inequality that is very similar to the QNEC in its relative entropy form (24). For continuous functions $C_1\ge C_2$ and $A\ge 0$, they showed

25 $$\begin{aligned} \left. \partial _t^+ S_{R(N_{C_1+tA})}(\Psi ||\Omega )\right| _{t=0}-\left. \partial _t^- S_{R(N_{C_2+tA})}(\Psi ||\Omega )\right| _{t=0}\ge 0, \end{aligned}$$

where $\partial _t^\pm$ denote half-sided partial derivatives. This statement was proven for the class of vectors satisfying the ANEC (22) with respect to $T_{++}$ and having finite relative entropy with respect to the vacuum and the algebra of the zero null cut.

We will make contact with the pyhsics literature and prove the ANEC for the free scalar field for certain states. Together with Proposition 4.1 and the results of [CF20], it proves the inequality (25). Moreover, we will verify the QNEC (24) for the free scalar field and for some states explicitly. In order to do this, we take the decomposition of the modular operator (cf. Theorem 4.3) and exploit the result by [Lon20] on each fiber. In this sense, our findings constitute a generalization of the results to a continuum family of *U*(1)-currents.

The ANEC To make contact with the physical literature, let us consider distorted lightlike translations $T_A$ by $A\ge 0$, which generate half-sided modular inclusions of standard subspaces $H(N_{C+A})\subset H(N_C)$ of null-cuts. It is claimed, e.g. [CTT17b,  (5.6) and (5.18)], that the following operators are positive.

26 $$\begin{aligned} P_{\pmb {x}_\perp }&=\int dx_+ T_{++}(x_+,\pmb {x}_\perp )\nonumber \\ H_A&=\int d^{D-1}\pmb {x}_\perp A(\pmb {x}_\perp )\int dx_+ T_{++}(x_+,\pmb {x}_\perp ) = \int ^\oplus _{{{\mathbb {R}}}^{D-1}} d^{D-1}\pmb {x}_\perp A(\pmb {x}_\perp )P_{\pmb {x}_\perp }. \end{aligned}$$

Furthermore, it is argued that positivity of $H_A$ should imply ANEC (22) in [FLPW16,  Section 3.2].

Note that the last expression does not involve $T_{++}$, and we can make sense of it in the free field if we interpret these relations at the one-particle level. Indeed, it is simply a weighted integral of the generator $P_{\pmb {x}_\perp }$ of translations in the $\mathrm{U(1)}$-current, which is positive.

Following [Lon20], we define the vacuum energy associated to null deformations by $A \ge 0$ of a normal and faithful state $\varphi$, that has a vector representative $\eta$, by

27 $$\begin{aligned} E_A(\eta )=\langle \eta ,H_{A}\eta \rangle , \end{aligned}$$

where $H_A=\frac{1}{2\pi }\big (\log (\Delta _{R(H(N_{C+A})),\Omega })$ $-\log (\Delta _{R(H(N_C)),\Omega })\big )$ is the generator of modular translations associated to the HSMI $R(H(N_{C+A}))\subset R(H(N_{C}))$. From Theorem 4.3, one concludes that the generator of modular translation coincides with (26).

The expression (27) is positive for the dense set of vectors in ${{\mathcal {D}}}(H_A)$ (cf. “Appendix A” for explicit form of ${{\mathcal {D}}}(H_A)$) since $H_A$ is a positive operator. In [Lon20] and [CF20], the positivity of (27) is considered as form of the ANEC.

The QNEC Let $\omega \circ \beta _{h_1}$ and $\omega \circ \beta _{h_2}$ be two coherent states that are unitarily generated by the adjoint action of $\mathrm w({\mathfrak {h}}_1)$ and $\mathrm w({\mathfrak {h}}_2)$, respectively. The relative entropy is between these states is:

$$\begin{aligned} S(\omega \circ \beta _{h_1}||\omega \circ \beta _{h_2})&=S(\omega \circ \beta _{h_1}||\omega \cdot {{\mathrm{Ad\,}}}\mathrm {w}({\mathfrak {h}}_2))=S(\omega \cdot {{\mathrm{Ad\,}}}\mathrm {w}({\mathfrak {h}}_1-{\mathfrak {h}}_2)||\omega )\\&=S(\omega \circ \beta _{h_1-h_2}||\omega ). \end{aligned}$$

To study the relative entropy between these states, we can therefore restrict to $h_2=0$, i.e. $\omega \circ \beta _{h_2}$ being the vacuum state.

A distorted lightlike translation $T_{tA}$ by continuous *A* maps $H(N_C)$ to $H(N_{C+tA})$ and the relative entropy of $\omega \circ \beta _h$ and $\omega$ changes accordingly (cf. Proposition 5.3). The differentiation with respect to the deformation parameter *t* gives:

28 $$\begin{aligned} \frac{d}{dt}S_{R(N_{C+tA})}(\omega \circ \beta _h||\omega )&=-\pi \int _{{{\mathbb {R}}}^{D-1}}\int _{C(\pmb {x}_\perp )+tA(\pmb {x}_\perp )}^\infty A(\pmb {x}_\perp )h(x_+,\pmb {x}_\perp )^2 dx_+d\pmb {x}_\perp \\ \frac{d^2}{dt^2}S_{R(N_{C+tA})} (\omega \circ \beta _h||\omega )&=\pi \int _{{{\mathbb {R}}}^{D-1}} A(\pmb {x}_\perp )^2 h(C(\pmb {x}_\perp )+tA(\pmb {x}_\perp ),\pmb {x}_\perp )^2 d\pmb {x}_\perp \ge 0 \nonumber \end{aligned}$$

We applied the differentiation fibrewise and used

29 $$\begin{aligned} \frac{d}{dt}\int _{C+tA(\pmb {x}_\perp )}^{\infty }(x_+-(C+tA(\pmb {x}_\perp )) h(x_+,\pmb {x}_\perp )^2 dx_+&=\int _{C+tA(\pmb {x}_\perp )}^\infty A(\pmb {x}_\perp )h(x_+,\pmb {x}_\perp )^2 dx_+ \end{aligned}$$

30 $$\begin{aligned} \frac{d^2}{dt^2}\int _{C+tA(\pmb {x}_\perp )}^\infty A(\pmb {x}_\perp )h(x_+,\pmb {x}_\perp )^2 dx_+&= A(\pmb {x}_\perp )^2 h(C+tA(\pmb {x}_\perp ),\pmb {x}_\perp )^2 . \end{aligned}$$

To justify the interchange of the $\pmb {x}_\perp$-integral and the $\frac{d}{dt}$-derivative, we verify that (29), (30) $\in L^1({{\mathbb {R}}}^{D-1},d^{D-1}\pmb {x}_\perp )$. We estimate (29) $\le \left\Vert A(\pmb {x}_\perp )h(x_+,\pmb {x}_\perp )^2\right\Vert _1$. Since *A* is continuous and *h* is compactly supported and smooth, the estimate is bounded and compactly supported in ${{\mathbb {R}}}^{D-1}$ and as such it is integrable. For (30), we note that $C+tA(\pmb {x}_\perp )$ is continuous on ${{\mathbb {R}}}^{D-1}$ and accordingly maps compact sets to compact sets. Hence, the product in (30) is compactly supported in ${{\mathbb {R}}}^{D-1}$ and bounded and therefore integrable.

In summary, we have proven the following form of the Quantum Null Energy Condition:

#### Corollary 5.4

For the coherent states $\omega \circ \beta _h$ considered here, the QNEC holds:

$$\begin{aligned} \frac{1}{2\pi }\frac{d^2}{dt^2}S_{R(N_{C+tA})} (\omega \circ \beta _h||\omega )\ge 0. \end{aligned}$$

This inequality is not saturated at every point of positive energy density. Following the arguments in Theorem 3.12 and Proposition 3.13, we can replace the region $N_{C+tA}$ with $N_{C+tA}''$ and $W_{C+tA}$, respectively.

As expected in [BFK+16], we recover the ANEC by integrating the QNEC along a null-direction for coherent states by (27). Here the ANEC has to be intended as the positivity of (27). We assume $A\ge 0$, then:

$$\begin{aligned}&\frac{1}{2\pi }\int _{{\mathbb {R}}}dt\frac{d^2}{dt^2}S_{R(N_{C+tA})} (\omega \circ \beta _h||\omega ) \\&\quad =\frac{1}{2}\int _{{\mathbb {R}}}dt \int _{{{\mathbb {R}}}^{D-1}}A(\pmb {x}_\perp )^2h(C(\pmb {x}_\perp )+tA(\pmb {x}_\perp ),\pmb {x}_\perp )^2d\pmb {x}_\perp \\&\quad =\frac{1}{2}\int _{{\mathbb {R}}}dx_+ \int _{{{\mathbb {R}}}^{D-1}}A(\pmb {x}_\perp )h(x_+,\pmb {x}_\perp )^2d\pmb {x}_\perp \\&\quad ={\mathrm {Im}\,\langle {\mathfrak {h}},iH_A{\mathfrak {h}} \rangle } = -i \left. \frac{d}{dt}\right| _{t=0}\langle {\mathfrak {h}},T_{tA}{\mathfrak {h}} \rangle \\&\quad =-i\left. \frac{d}{dt}\right| _{t=0}\left( e^{-\frac{1}{2}(\left\Vert {\mathfrak {h}}\right\Vert ^2+\left\Vert T_{tA}{\mathfrak {h}}\right\Vert ^2)} e^{\langle {\mathfrak {h}},T_{tA}{\mathfrak {h}} \rangle } \right) = -i\left. \frac{d}{dt}\right| _{t=0}\langle \mathrm w({\mathfrak {h}})\Omega ,\Gamma (T_{tA})\mathrm w({\mathfrak {h}})\Omega \rangle \\&\quad =\langle \mathrm w({\mathfrak {h}})\Omega ,d\Gamma (H_A) \mathrm w({\mathfrak {h}})\Omega \rangle , \end{aligned}$$

where the self-adjoint fibrewise “momentum operator" $H_A=\int _{{{\mathbb {R}}}^{D-1}}^\oplus A(\pmb {x}_\perp )P_{\pmb {x}_\perp }d\pmb {x}_\perp$ is the generator of distorted lightlike translations $T_{tA}$, $d\Gamma (H_A)$ is the additive second quantization of $H_A$, i.e. the generator of $\Gamma (T_{tA})$. (7) and (21) are used to conclude the third equality and the general identity (6) is used for the fifth equality. This is in agreement with [Lon20,  Corollary 3.10 and (46)]).

We interpret $A(\pmb {x}_\perp )h(x_+,\pmb {x}_\perp )^2$ as vacuum energy density at the point $(x_+,\pmb {x}_\perp ) \subset X_-^0$ of the state $\mathrm w({\mathfrak {h}}) \Omega$ with respect to null-deformations *A* . This is the vacuum energy density of the state $\mathrm w({\mathfrak {h}}(\pmb {x}_\perp ))\Omega _{\pmb {x}_\perp }$ of the *U*(1)-current at $\pmb {x}_\perp$-fibre with respect to deformations by $A(\pmb {x}_\perp )$ (cf. [Lon20]). With this interpretation, the energy averaged over the a null-cut $N_C$ is:

31 $$\begin{aligned} E_{A,\beta _h}( N_C)=\int _{{{\mathbb {R}}}^{D-1}}\int _{C(\pmb {x}_\perp )}^\infty A(\pmb {x}_\perp )h(x_+,\pmb {x}_\perp )^2d\pmb {x}_\perp , \end{aligned}$$

and we have the following connection between the first derivative of the relative entropy and the energy localised in the null-cut by comparing (28) and (31):

$$\begin{aligned} \frac{d}{dt}S_{R(N_{C+tA})}(\omega \circ \beta _h||\omega )=E_{A,\beta _h}(N_{C+tA}). \end{aligned}$$

Strong superadditivity of relative entropy Consider two null-cuts $N_{C_1}$ and $N_{C_2}$ and the null-cuts $N_{C_\cup }$ and $N_{C_\cap }$ generated by $C_\cup (\pmb {x}_\perp )=\min \{C_1(\pmb {x}_\perp ),C_2(\pmb {x}_\perp )\}$ and $C_\cap (\pmb {x}_\perp )=\max \{C_1(\pmb {x}_\perp ),C_2(\pmb {x}_\perp )\}$, respectively. Then the strong superadditivity of relative entropy is:

$$\begin{aligned} S_{R(N_{C_\cup })}(\Psi ||\Omega ) + S_{R(N_{C_\cap })}(\Psi ||\Omega ) \ge S_{R(N_{C_1})}(\Psi ||\Omega ) + S_{R(N_{C_2})}(\Psi ||\Omega ). \end{aligned}$$

As proven in [CF20,  Section 3.2] the QNEC (25) implies this strong superadditivity for a state $\Psi$ with finite QNEC (25). We can show that the states considered in Sect. 5.3 saturate the strong superadditivity of relative entropy. Indeed, we apply Proposition 5.3 and have:

$$\begin{aligned}&S_{R(H(N_{C_\cup }))}(\omega \circ \beta _h||\omega )+S_{R(H(N_{C_\cap }))}(\omega \circ \beta _h||\omega )-S_{R(H(N_{C_1}))}(\omega \circ \beta _h||\omega )-S_{R(H(N_{C_2}))}(\omega \circ \beta _h||\omega )\\&\quad =\pi \int _{{{\mathbb {R}}}^{D-1}}\Big (\int _{C_\cup (\pmb {x}_\perp )}^\infty (x_+-C_\cup (\pmb {x}_\perp )) +\int _{C_\cap (\pmb {x}_\perp )}^\infty (x_+-C_\cap (\pmb {x}_\perp ))\\&\qquad -\int _{C_1(\pmb {x}_\perp )}^\infty (x_+-C_1(\pmb {x}_\perp ))-\int _{C_2(\pmb {x}_\perp )}^\infty (x_+-C_2(\pmb {x}_\perp )) \Big ) h(x_+,\pmb {x}_\perp )^2 dx_+ d\pmb {x}_\perp =0 \end{aligned}$$

since $C_\cup (\pmb {x}_\perp )+C_\cap (\pmb {x}_\perp )-C_1(\pmb {x}_\perp )-C_2(\pmb {x}_\perp )=0$ for all $\pmb {x}_\perp$.

## Concluding Remarks

Let us close this paper with a few more remarks.

- It is possible to extend the definition of local subspaces on the null plane to measurable functions *C*. The strategy is to loosen the assumption on “thin test functions” (cf. Sect. 3.3) in the sense that $\pmb {x}_\perp \mapsto g(x_+,\pmb {x}_\perp ) \in L^1({{\mathbb {R}}}^{D-1})\cap L^2({{\mathbb {R}}}^{D-1})$ for almost every $x_+$. The real subspaces are covariant with respect to distorted lightlike translations and dilations (cf. (15) and (16)) for measurable functions *C*. Also in this case $T_C$ and $D_C$ are determined by measurable vector fields of bounded operators since Remark 3.9 obviously extends. It constitutes an extension of the present analysis in the sense that for continuous functions the definitions coincide. Many results such as the decomposition of the subspaces (Proposition 3.11) and modular operators (Theorem 4.3) hold for the resulting subspaces as well. However, isotony (Lemma 3.7) and duality for null-cuts (Theorem 3.12) do not hold for arbitrary measurable functions.
- The modular operator of a direct integral of standard subspaces decomposes into the direct integral of modular operators. Therefore, the modular operator of *H*(*R*) from Proposition 3.11 decomposes into the direct integral of *U*(1)-modular operators of the interval $(C_1(\pmb {x}_\perp ),C_2(\pmb {x}_\perp ))$.
- The fact that there are observables that can be restricted to null plane shows that the minimal localization region in the sense of [Kuc00] can have empty interior.
- In a general Haag–Kastler net, we do not expect that there are sufficiently many observables (e.g. in the sense of the Reeh-Schlieder property) that can be restricted to the null plane. With additional assumptions (including the ${{\mathbb {C}}}$-number commutation relations), it is shown that only free fields can be directly restricted on the null plane [Dri77a], cf. [Wal12,  Section 5] [BCFM15,  A]. In addition, in the two-dimensional spacetime, there are interacting Haag–Kastler nets [Tan14] where observables on the lightray generate a proper subspace of the Hilbert space from the vacuum [BT15,  Section 5.3, Trivial examples]. In these cases, different ideas are required to justify (1). On the other hand, it has been suggested that there could be bounded operators localized on the null plane in the sense of Haag duality [Sch05]. If so, two-dimensional conformal field theory is hidden on each lightlike fibre [BLM11].

## Decomposable Functional Calculus

Assume the same notations as in Sect. 3.1. We call an (possibly unbounded) operator *T* on the direct integral of Hilbert spaces ${{\mathcal {K}}}=\int ^\oplus _X {{\mathcal {K}}}_\lambda d\nu (\lambda )$ decomposable when there is a function $\lambda \mapsto T_\lambda$, such that $T_\lambda$ is an (possibly unbounded) operator on ${{\mathcal {K}}}_\lambda$, and for each $\xi \in {{\mathcal {D}}}(T)$, $\xi (\lambda ) \in {{\mathcal {D}}}(T_\lambda )$ and $(T\xi )(\lambda )=T_\lambda \xi (\lambda )$ for $\nu$-almost every $\lambda$.

### Theorem A.1

Let *T* be a self-adjoint operator on the direct integral of Hilbert spaces ${{\mathcal {K}}}=\int ^\oplus _X K_\lambda d\nu (\lambda )$. The following are equivalent:

1. The projection-valued measure $E^T$ associated to *T* decomposes in the direct integral of projection-valued measures:
  32 $$\begin{aligned} E^T=\int ^\oplus _X E^{T_\lambda }d\nu (\lambda )=:E \end{aligned}$$
  for some self-adjoint operators $T_\lambda$ on ${{\mathcal {K}}}_\lambda$.
2. The Borel functional calculus of *T* decomposes in the direct sum of Borel functional calculi of self-adjoint operators $T_\lambda$ on ${{\mathcal {K}}}_\lambda$:
  $$\begin{aligned} f(T)=\int ^\oplus _X f(T_\lambda )d\nu (\lambda ). \end{aligned}$$
3. $T=\int ^\oplus _X T_\lambda d\nu (\lambda )$ is decomposable. Each $T_\lambda$ is a self-adjoint operator on ${{\mathcal {K}}}_\lambda$.
4. The one-parameter group of unitaries $U(t):=e^{itT}$ decomposes:
  $$\begin{aligned} U(t)=\int ^\oplus _X U_\lambda (t)d\nu (\lambda ), \end{aligned}$$
  where $U_\lambda (t)$ is a one-parameter group of unitaries on ${{\mathcal {K}}}_\lambda$.

### Proof

$$2 \Rightarrow 3, 4$$: clear.

$3 \Leftrightarrow 1 \Rightarrow 2$: We show that *E* (cf. (32)) is the spectral measure associated to $\int ^\oplus _X T_\lambda d\nu (\lambda )$. It is a projection-valued measure defined by:

$$\begin{aligned} \Omega \mapsto E(\Omega ):=\int ^\oplus _X E^{T_\lambda }(\Omega )d\nu (\lambda ). \end{aligned}$$

That is because the multiplication of decomposable operators is defined fibre-wise. It is clear that each element is a projection and that $E(\emptyset )=0$, $E({{\mathbb {R}}})=1$ and $E(\Omega _1 \cap \Omega _2)=E(\Omega _1)E(\Omega _2)$. For mutually disjoint $\Omega _n$ the associated projections are orthogonal. It follows that their sum is again a projection. Hence, we can use dominated convergence, to deduce the convergence $E(\cup ^N \Omega _n)\xrightarrow {N\rightarrow \infty }E(\Omega )$ for a countable union $\Omega =\cup \Omega _n$ from the fibre-wise convergence. The latter is true because $E^{T_\lambda }$ is a spectral measure. The spectral measure $E_\varphi$ for $\varphi \in {{\mathcal {K}}}$ is for a Borel set $\Omega$:

$$\begin{aligned} E_\varphi (\Omega )=\langle \varphi ,E(\Omega )\varphi \rangle&=\int _X E^{T_\lambda }_{\varphi _\lambda }(\Omega ) d\nu (\lambda ). \end{aligned}$$

For integration, we denote it by $dE_\varphi (x)$. For a simple function $s=\sum a_i \chi _{\Omega _i}$ on a compact set $\Omega =\cup _{i} \Omega _i$ it holds:

$$\begin{aligned} \int _{{\mathbb {R}}}s(x) dE_\varphi (x)&=\sum \limits _{i}a_i\int _X E_{\varphi _\lambda }^{T_\lambda }(\Omega _i)d\nu (\lambda )\\&=\int _X \int _{{\mathbb {R}}}s(x) dE^{T_\lambda }_{\varphi _\lambda }(x) d\nu (\lambda )=\langle \varphi ,\left( \int ^\oplus _X s(T_\lambda )d\nu (\lambda )\right) \varphi \rangle . \end{aligned}$$

The integral of a general bounded Borel function is the difference of the integrals over the positive and negative part. The integral over a positive bounded Borel function *f* with respect to $dE_\varphi$ is defined as the supremum of integral over simple functions *s* that are locally bounded by *f*:

$$\begin{aligned} \int _{{\mathbb {R}}}f(x) dE_\varphi (x)&=\sup \limits _{0\le s\le f}\int _X \int _{{\mathbb {R}}}s(x) dE^{T_\lambda }_{\varphi _\lambda }(x) d\nu (\lambda )\le \int _X \sup \limits _{0\le s\le f}\int _{{\mathbb {R}}}s(x) dE^{T_\lambda }_{\varphi _\lambda }(x) d\nu (\lambda ) \end{aligned}$$

For a positive, Borel measurable function *f*, there is a non-decreasing sequence $s_n$ of simple functions converging from below pointwise. We can utilize dominated convergence (spectral measures are finite) to show

$$\begin{aligned} \lim \limits _{n\rightarrow \infty }\int _X \int _{{\mathbb {R}}}s_n(x) dE_{\varphi _\lambda }(x) d\nu (\lambda )=\int _X \int _{{\mathbb {R}}}f(x) dE_{\varphi _\lambda }(x) d\nu (\lambda ). \end{aligned}$$

Hence, we have for bounded Borel functions:

33 $$\begin{aligned} \int _X \int _{{\mathbb {R}}}f(x) dE^{T_\lambda }_{\varphi _\lambda }(x) d\nu (\lambda ) =\int _{{\mathbb {R}}}f(x) dE_\varphi (x). \end{aligned}$$

For each $\varphi \in {{\mathcal {K}}}$, the measure $E_\varphi$ is inner regular and countably additive. The second property was already discussed. The inner regularity follows from the finiteness of $E_\varphi$. Hence, the two integrals in (33) define the same bounded, linear functional on the compactly supported, continuous functions. From the uniqueness in the Riesz-Markov theorem (cf. Lemma A.2), we deduce the equality of the measures. This means for each $\varphi \in {{\mathcal {K}}}$ and Borel function *h*:

$$\begin{aligned} \int _{{\mathbb {R}}}h(x)dE_\varphi =\int _X\int _{{\mathbb {R}}}h(x)dE_{\varphi _\lambda }^{T_\lambda }d\nu (\lambda )=\langle \varphi , \left( \int ^\oplus _X h(T_\lambda )d\nu (\lambda ) \right) \varphi \rangle . \end{aligned}$$

In the usual notation, this amounts to:

$$\begin{aligned} \int _{{\mathbb {R}}}h(x)dE(x)=\int ^\oplus _X h(T_\lambda ) d\nu (\lambda ). \end{aligned}$$

The domain of the operator *h*(*T*) is:

$$\begin{aligned} {{\mathcal {D}}}(h(T))&=\{\varphi \in {{\mathcal {K}}}; \int _{{\mathbb {R}}}|f(k)|^2 dE_{\varphi }^T(k)<\infty \}\\&=\{\varphi \in {{\mathcal {K}}}; \int _X\int _{{\mathbb {R}}}|f(k)|^2 dE_{\varphi (\lambda )}^{T_\lambda }(k)d\nu (\lambda )<\infty \}\\&= \{\varphi \in {{\mathcal {K}}}; \int _X\left\Vert h(T_\lambda )\varphi (\lambda )\right\Vert ^2 d\nu (\lambda )<\infty \} =\int ^\oplus _X {{\mathcal {D}}}(h(T_\lambda ))d\nu (\lambda ). \end{aligned}$$

For a closed operator *A* on a Hilbert space ${{\mathcal {H}}}$ its domain ${{\mathcal {D}}}(A)$ is a Hilbert space w.r.t. the Graph inner product:

$$\begin{aligned} \langle \xi ,\eta \rangle _{A}:=\langle \xi ,\eta \rangle _{{{\mathcal {H}}}}+\langle A\xi ,A\eta \rangle _{{{\mathcal {H}}}}. \end{aligned}$$

Since $T_\lambda$ is self-adjoint for ($\nu$-almost) every $\lambda$, the domain of $h(T_\lambda )$ is a Hilbert space w.r.t. the Graph inner product for almost every $\lambda$. The domain ${{\mathcal {D}}}(h(T))$ embeds in ${{\mathcal {K}}}$ trivially. If *T* is decomposable, then we have for all vectors $\varphi \in {{\mathcal {K}}}$:

$$\begin{aligned} \int _{{\mathbb {R}}}\lambda dE_\varphi ^T(\lambda )=\int _X\int _{{\mathbb {R}}}\lambda dE_{\varphi _\lambda }^{T_\lambda }d\nu (\lambda ). \end{aligned}$$

$4\Rightarrow 1$: Let $\xi , \eta \in {{\mathcal {K}}}$ and $g\in {\mathscr {S}}({{\mathbb {R}}})$. Then by Fubini’s theorem the operator *g*(*T*) decomposes:

$$\begin{aligned} \langle \xi , g(T)\eta \rangle&=\int _{{\mathbb {R}}}{\tilde{g}}(t)\langle \xi ,U(t)\eta \rangle dt\\&=\int _X \langle \xi (\lambda ),\left( \int _{{\mathbb {R}}}{\tilde{g}}(t)U_\lambda (t)dt \right) \eta (\lambda ) \rangle d\nu (\lambda )\\&=\langle \xi ,\left( \int ^{\oplus }_X g(T_\lambda )d\nu (\lambda ) \right) \eta \rangle . \end{aligned}$$

Every characteristic function $\chi _S$ of a set *S* with finite measure can be expressed as the pointwise limit of a sequence $g_n\subset {\mathscr {S}}({{\mathbb {R}}})$ of uniformly bounded functions. In the functional calculus this amounts to the strong convergence:

$$\begin{aligned} g_n(T)\xrightarrow {n\rightarrow \infty }\chi _{S}(T)=E^T(S), \end{aligned}$$

where $E^T(S)$ denotes the spectral projection of *T* with respect to the set *S*.

Since $g_n$ is uniformly bounded, we can apply the theorem of dominated convergence:

$$\begin{aligned} \langle \xi ,E^T_{S}\eta \rangle&=\lim \limits _{n\rightarrow \infty }\int _X\langle \xi (\lambda ),g_n(T_\lambda )\eta (\lambda ) \rangle d\nu (\lambda )\\&=\int _X\langle \xi (\lambda ),\chi _{S}(T_\lambda )\eta (\lambda ) \rangle d\nu (\lambda )\\&=\langle \xi ,\left( \int ^\oplus _X E^{T_\lambda }(S)d\nu (\lambda )\right) \eta \rangle . \end{aligned}$$

Hence, the spectral measure $E_{\varphi }^{T}$ associated to any vector $\varphi \in {{\mathcal {K}}}$ decomposes in the following sense for any bounded Borel function *f*:

34 $$\begin{aligned} \langle \varphi ,f(T)\varphi \rangle&=\int _{{\mathbb {R}}}f(k) dE_{\varphi }^T(k) \end{aligned}$$

35 $$\begin{aligned}&=\int _X\int _{{\mathbb {R}}}f(k) dE_{\varphi (\lambda )}^{T_\lambda }(k) =\langle \varphi ,\left( \int ^\oplus _X f(T_\lambda )d\nu (\lambda )\right) \varphi \rangle . \end{aligned}$$

The two expression (34) and (35) define the same linear functional on the continuous, compactly supported functions. By the Riesz-Markov theorem such a functional is determined a unique countably additive, inner regular measure on ${{\mathbb {R}}}$. Since both measure in (34) and (35) are countably additive and inner regular, they coincide. $\square$

### Lemma A.2

(Riesz-Markov theorem). Let *X* be a locally compact Hausdorff space and $\Psi$ a bounded linear functional on ${{\mathcal {C}}}_c(X)$, then there exists a unique inner regular and countably additive measure $\mu$ on *X* such that:

$$\begin{aligned} \Psi (f)=\int _X f d\mu . \end{aligned}$$

## Footnotes

1 With this slightly unfortunate notations, $W_1$ is the standard wedge in the $x_1$-direction (and not the shifted wedge).
